# Reconciling Local Coupled Cluster with Multireference Approaches for Transition Metal Spin-State Energetics

**DOI:** 10.1021/acs.jctc.2c00265

**Published:** 2022-05-18

**Authors:** Maria Drosou, Christiana A. Mitsopoulou, Dimitrios A. Pantazis

**Affiliations:** † Inorganic Chemistry Laboratory, National and Kapodistrian University of Athens, Panepistimiopolis, Zografou 15771, Greece; ‡ Max-Planck-Institut für Kohlenforschung, Kaiser-Wilhelm-Platz 1, 45470 Mülheim an der Ruhr, Germany

## Abstract

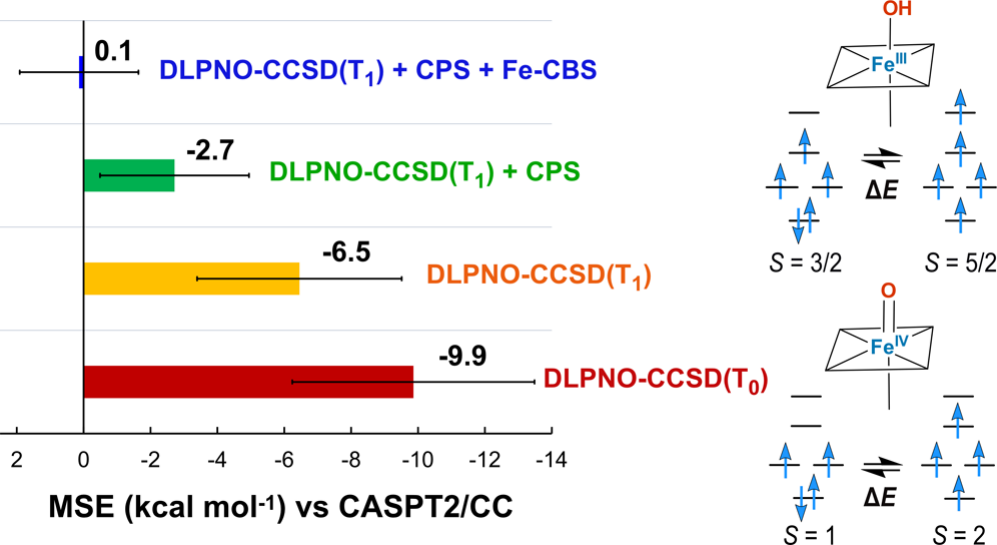

Spin-state energetics of transition metal complexes remain one of the most challenging targets for electronic structure methods. Among single-reference wave function approaches, local correlation approximations to coupled cluster theory, most notably the domain-based local pair natural orbital (DLPNO) approach, hold the promise of bringing the accuracy of coupled cluster theory with single, double, and perturbative triple excitations, CCSD(T), to molecular systems of realistic size with acceptable computational cost. However, recent studies on spin-state energetics of iron-containing systems raised doubts about the ability of the DLPNO approach to adequately and systematically approximate energetics obtained by the reference-quality complete active space second-order perturbation theory with coupled-cluster semicore correlation, CASPT2/CC. Here, we revisit this problem using a diverse set of iron complexes and examine several aspects of the application of the DLPNO approach. We show that DLPNO-CCSD(T) can accurately reproduce both CASPT2/CC and canonical CCSD(T) results if two basic principles are followed. These include the consistent use of the improved iterative (T_1_) versus the semicanonical perturbative triple corrections and, most importantly, a simple two-point extrapolation to the PNO space limit. The latter practically eliminates errors arising from the default truncation of electron-pair correlation spaces and should be viewed as standard practice in applications of the method to transition metal spin-state energetics. Our results show that reference-quality results can be readily achieved with DLPNO-CCSD(T) if these principles are followed. This is important also in view of the applicability of the method to larger single-reference systems and multinuclear clusters, whose treatment of dynamic correlation would be challenging for multireference-based approaches.

## 1 Introduction

The development of accurate, efficient, and universal approaches for computing energy gaps between different spin states in transition metal complexes persists as a major challenge for quantum chemistry.^1−6^ Significant applications that rely on accurate spin-state energetics include molecules and materials with specific magnetic properties, where reliable prediction of the ground and excited spin states and possible spin-crossover (SCO) behavior^7−9^ is a prerequisite for rational design, and deciphering multistate reactivity that is often crucial in bioinorganic chemistry and catalysis.^10−12^ Although corrections for environmental and thermal effects may be important additional considerations for certain applications, the principal obstacle for all quantum chemical methodologies remains the accurate calculation of electronic energy differences between spin states.

Extensive experience with density functional theory (DFT) has established that the calculated relative energies between species with different numbers of unpaired electrons—or with the same electronic configuration but different spin coupling in the case of exchange-coupled systems—depend strongly on the choice of approximate exchange-correlation functionals.^13−21^ The Hartree–Fock (HF) wavefunction includes Fermi but not Coulomb correlation; therefore, the HF method is strongly biased toward high-spin (HS) states, whereas the local density approximation and generalized gradient approximation functionals overstabilize delocalized charge distributions, introducing a bias toward low-spin (LS) states.^18,20^ Mixing the two components in hybrid DFT methods can lead to sufficiently systematic error cancellation, so that “optimal” hybrid DFT methods have been proposed for specific classes of transition metal complexes.^22−32^ Approaches that combine DFT with wavefunction-based methods also show promising results.^16,33−36^ However, it remains hard to know a priori the best approach for a system and impossible to define a universally applicable DFT method for spin states, while the limitations of this semiempirical tailoring approach are laid bare when one considers molecular properties beyond energetics.

Wave function theory (WFT) methods are the obvious alternative^37−48^ because they attempt to approximate the full correlation energy in a systematically improvable way. The coupled cluster with singles, doubles, and perturbative triples, CCSD(T),^49−51^ is known as the “gold standard”, at least for systems that are not strongly multiconfigurational.^41,42^ Nontrivial transition metal complexes of realistic size can only be treated with approximate WFT approaches, which means that recovery of 100% of the correlation energy remains impossible. Therefore, the emphasis in the case of spin-state energetics is on the balanced description of the correlation energy for different spin states. Local coupled cluster approaches offer the most promising route forward in this respect. Among the various local correlation approaches, a technique that has gained prominence in recent years is the domain-based local pair natural orbital (DLPNO) approach,^52−55^ which offers near-linear scaling and accuracy that can be systematically converged toward canonical CCSD(T) via a simple set of parameters.^56,57^ DLPNO-CCSD(T) has already been used successfully to describe large bioinorganic^58−61^ and other open-shell systems.^62−70^

This approach has also been used for the exceptionally hard problem of spin-state energetics in iron complexes.^57,71−75^ They represent a particularly challenging category of system, that is why they are often used as the “ultimate” testing ground for quantum chemical methods. The combination of complete active space second-order perturbation theory (CASPT2) for valence correlation with coupled-cluster semicore correlation (CASPT2/CC) was proposed^40^ as the most accurate method in this case. This results from the observation^76^ that the systematic overstabilization of HS states with respect to CCSD(T) references derives from inaccurate description of 3s3p correlation, which is therefore treated by coupled cluster theory in the compound approach. CASPT2/CC is thus considered to provide reference values for spin-state energetics of transition metal complexes, while full CCSD(T) may be considered superior to CASPT2/CC for the description of the low-lying spin states dominated by a single electronic configuration.^40^ It was precisely based on comparison with high-quality CASPT2/CC results on spin-state energetics of iron complexes that local coupled cluster methods were deemed to have severe limitations in their performance.^73−75^ Specifically, it was reported that DLPNO-CCSD(T) systematically overstabilizes HS states for quintet–triplet gaps of Fe(IV)–oxo complexes by around 7–10 kcal mol^–1^,^74^ which was attributed mostly to the contribution of single and double excitations.^75^

Clearly, errors of this magnitude for mononuclear complexes would imply that local correlation approaches in general and the popular DLPNO approach in particular may be of limited utility for spin-state energetics of electronically challenging open-shell systems. In the present study, we look into this problem with greater detail and we reach much more encouraging conclusions. We investigate the spin-state energetics of a varied set of twelve iron complexes using specific operational protocols that we show to be essential in applications of the DLPNO-CCSD(T) approach. Specifically, we apply a DLPNO-CCSD(T) protocol that involves both complete PNO space (CPS) extrapolation, as recently introduced by Altun et al.,^77^ and complete basis set (CBS) limit extrapolation with respect to the iron site. Comparison of the results with CASPT2/CC reference values shows that the obtained DLPNO-CCSD(T) values are practically equivalent to CASPT2/CC. Overall, even for the demanding case of iron complexes, DLPNO-CCSD(T) is able to accurately and systematically reproduce CASPT2/CC and canonical CCSD(T) spin-state energetics while retaining its practical benefits of ease-of-use, efficiency, and scalability.

## 2 Methodology

### 2.1 Set of Complexes

**Figure 1:**
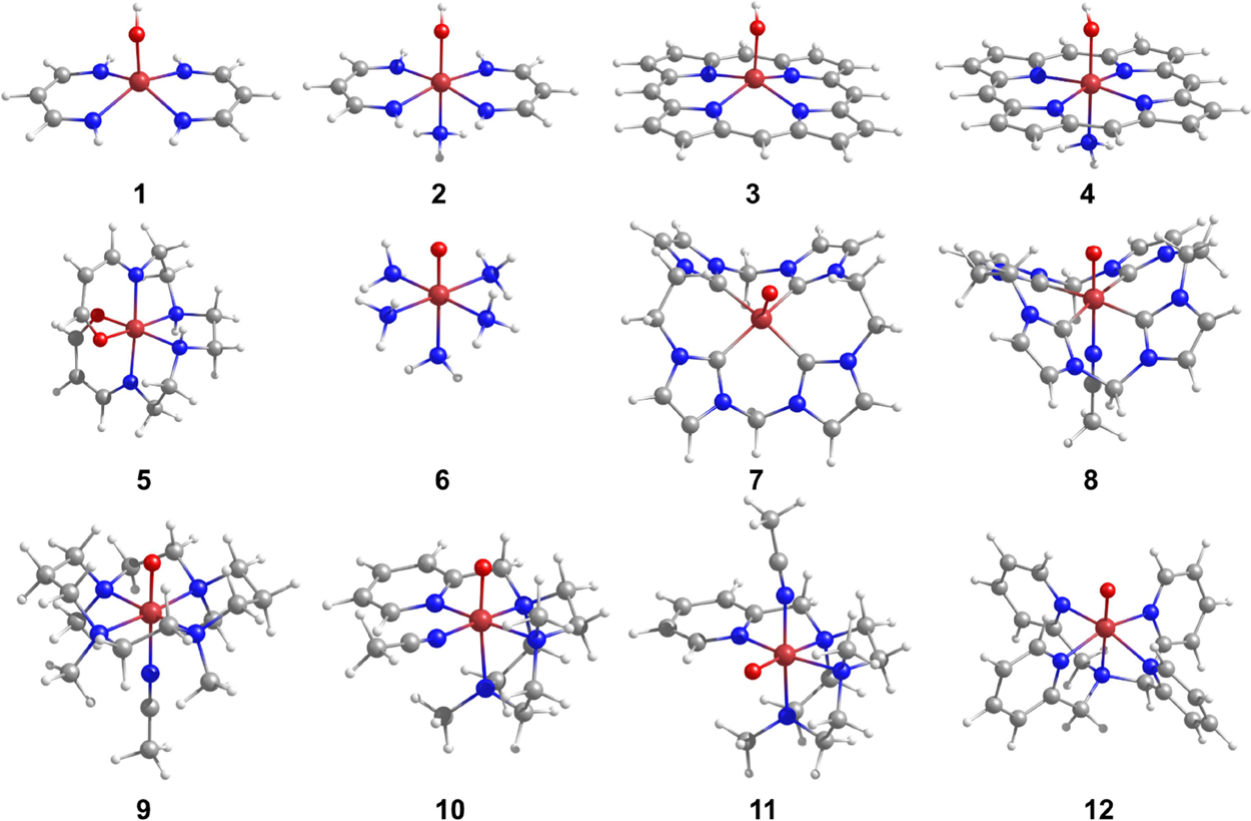
Molecular structures of the 12 iron complexes investigated in this work. Ligand abbreviations; (**1**) [Fe^III^L_2_OH], L = propyl-amidine, (**2**) [Fe^III^L_2_(NH_3_)(OH)], (**3**) [Fe^III^P(OH)], P = porphyrin, (**4**) [Fe^III^P(OH)(NH_3_)], (**5**) [Fe^III^(acac_2_trien)]^+^, H_2_acac_2_trien = Schiff base obtained from the 1:2 condensation of triethylenetetramine with acetylacetone, (**6**) [Fe^IV^(O)(NH_3_)_5_]^2+^, (**7**) [Fe^IV^(O)(NHC)]^2+^, NHC = 3,9,14,20-tetraaza-1,6,12,17-tetraazoniapenta-cyclohexacosane-1(23),4,6(26),10,12(25),15,17(24),21-octaene, (**8**) [Fe^IV^(O)(NHC)(MeCN)]^2+^, (**9**) [Fe^IV^(O)(TMC)(MeCN)]^2+^, TMC = 1,4,8,11-tetramethyl-1,4,8,11-tetraazacyclotetradecane, (**10**) [Fe^IV^(*O*)^ax^(PyTACN)(MeCN)]^2+^, PyTACN = 1-[2′-(pyridyl)-methyl]-4,7-dimethyl-1,4,7-triazacyclononane, (**11**) [Fe^IV^(*O*)^eq^(PyTACN)(MeCN)]^2+^, [Fe^IV^(O)(NHC)(MeCN)]^2+^, and (**12**) [Fe^IV^(O)(N4Py)]^2+^, N4Py = *N*,*N*-bis(2- pyridylmethyl)bis(2-pyridyl)methylamine).

We selected a varied set of twelve iron complexes, which include five Fe(III)–hydroxo and seven Fe(IV)–oxo complexes with different ligand field strengths, spin multiplicities, and range of electronic energy differences between the high- and low-spin isomers. The structures are shown in Figure 1.

Spin-state relative energies, i.e., spin-state splittings, in this work are expressed as
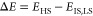
1where HS indicates the high spin state, IS and LS indicate the intermediate spin state and low spin state, respectively, and *E* is the electronic energy component. For Fe(III) complexes **1**–**5**, the HS states are sextets (*S* = 2.5), the IS states are quartets (*S* = 1.5), and the LS states are doublets (*S* = 0.5). For Fe(IV) complexes **6**–**12**, the HS states are quintets (*S* = 2) and the LS states are triplets (*S* = 1). In this work, adiabatic spin-state splittings are examined, meaning that the electronic energy *E* of each spin state is calculated using the structure that is optimized for the specific spin state. Therefore, Δ*E* indicates the electronic energy difference between minima of the potential energy surfaces of each spin state.

Structure coordinates of complexes **1**–**4** were taken from reference (40), where they were optimized separately for each spin state at the BP86/def2-TZVP level, of complex **5** from ref (41) optimized at the BP86-D3/def2-TZVP level, and of complexes **6**–**12** from ref (74) optimized at the BP86-D3BJ/def2-TZVP level. The reference CASPT2/CC-calculated Δ*E* values for each structure were obtained from the corresponding papers. Hence, the DLPNO-CCSD(T) calculations presented here have been performed on the same structures as the CASPT2/CC calculations that are used as the reference. The differences, ΔΔ*E*, of the DLPNO-CCSD(T)-calculated spin-state splittings, Δ*E*, from the reported CASPT2/CC calculated values are expressed as ΔΔ*E* = Δ*E*^DLPNO-CCSD(T)^ – Δ*E*^CASPT2/CC^.

### 2.2 Computational Details

All DLPNO-coupled cluster calculations were performed with Orca 5.^78^ Perturbative triple excitations were treated both with the semicanonical (T_0_)^53^ and the improved iterative (T_1_)^55^ approximations, and the differences among the two methods are discussed in detail. Subvalence correlation effects were accounted for using the default frozen core settings for Orca 5.^79^ For Fe, the 3s and 3p core orbitals were included in the correlation treatment, while the 1s and 2p electrons were kept frozen. For all other atoms except H, only the core 1s electrons were kept frozen. Quasi-restricted orbitals generated from unrestricted Kohn–Sham calculations were used to construct the reference determinant. The spin contamination values for the UKS orbitals are given in Table S14. For the DFT calculations, tight energy convergence criteria were used. Since relativistic effects have been reported to show non-negligible contributions to spin-state energetic calculations,^57,80^ scalar relativistic effects were considered throughout via the use of the zero-order regular approximation (ZORA),^81−83^ combined with ZORA-recontracted^84^ versions of the def2 basis sets.^85^ Two basis set combinations were employed. The first basis set combination is denoted as TZ/TZ, where the ZORA-def2-TZVPP was used for Fe, ZORA-def2-TZVP on O, C, and N, and ZORA-def2-SVP on H. The corresponding auxiliary basis sets def2-TZVPP/C on Fe, def2-TZVP/C on O, C, and N, and def2-SVP/C on H were used. The second basis set combination is denoted as QZ/TZ, where the ZORA-def2-QZVPP was used for Fe, ZORA-def2-TZVP on O, C, and N, and ZORA-def2-SVP on H along with the respective auxiliary basis sets.

Two-point extrapolation to the CBS limit with respect to Fe was carried out, while the ligand basis set was kept fixed. Fe CBS limit extrapolations for the self-consistent field and correlation energy parts, respectively, were performed according to the following equations:
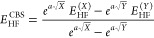
2
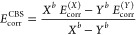
3where *a* = 7.88, *b* = 2.97, and *X* and *Y* are the two basis set hierarchies, *X* = 3 for TZ/TZ, and *Y* = 4 for QZ/TZ basis sets.^86^ The CBS extrapolation in eq 3 was applied only to DLPNO-CCSD(T) results with the default NormalPNO settings (Table 1) to yield an additive correction term, *δ*^CBS^, which is defined as
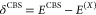
4

**Table 1:** Values of the *T*_CutPairs_, *T*_CutPNO,_ and *T*_CutDO_ Thresholds for the Three Default DLPNO Settings in Orca

default settings	*T*_CutPairs_	*T*_CutPNO_	*T*_CutDO_
tightPNO	10^–5^	1.00 × 10^–7^	5 × 10^–3^
normalPNO	10^–4^	3.33 × 10^–7^	1 × 10^–2^
loosePNO	10^–3^	1.00 × 10^–6^	2 × 10^–2^

The two key cutoff parameters that control the size of the correlation space in the DLPNO approach, i.e., the level of approximation of the method, are *T*_CutPairs_ and *T*_CutPNO_. Electron pairs with estimated pair correlation energies that are above the *T*_CutPairs_ parameter are classified as “strong pairs” and treated with the canonical coupled cluster, while for the remaining “weak pairs”, the local MP2 correlation energy is used. PNOs with occupation numbers smaller than the *T*_CutPNO_ parameter will be neglected for the respective electron pair; hence, the *T*_CutPNO_ cutoff determines the size of the correlation space for each electron pair.^52,87^ In Orca, three default sets of collective cutoff parameters for DLPNO-CCSD(T) calculations have been optimized, which control the level and accuracy of the approximation. The respective parameters are shown in Table 1. *T*_CutDO_ controls the size of the domains expanding the PNOs in terms of the pair atomic orbitals.^52^ TightPNO settings offer the highest accuracy, while LoosePNO settings are suggested only for rapid estimates. In this work, DLPNO-CCSD(T_1_) calculations were performed on the basis of NormalPNO and TightPNO settings.

The two-point PNO extrapolation method, proposed by Altun et al.,^77^ involves extrapolation of the correlation energies obtained using two different *T*_CutPNO_ cutoff values, using parameters derived from extensive benchmarking, in order to approach the CPS limit. They showed that this method also decreases the system size dependence of the DLPNO error.^88^ We performed two-point extrapolation of the correlation energies, *E^x^* and *E^y^*, calculated with different *T*_CutPNO_ thresholds, *T*_CutPNO_ 1.0 × 10^–*x*^ and 1.0 × 10^–*y*^, respectively, according to the following equation:^77^
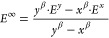
5where *E*^∞^is the correlation energy at the CPS limit and β is a constant. β can be integrated in a parameter *F* that also depends on the *T*_CutPNO_ thresholds as follows:
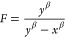
6and therefore eq 5 can be written as
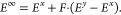
7

The optimal value of parameter *F* that minimizes the DLPNO error relative to canonical CCSD(T) was reported to be within the 1.5 ± 0.2 range for extrapolations with (*x*,*y*) values of (5,6) and (6,7).^77^

Two types of CPS extrapolation were used in this work. The first is denoted as CPS1 and was performed using DLPNO-CCSD(T_1_) correlation energies obtained with two different *T*_CutPNO_ values, 1.00 × 10^–6^ and 3.33 × 10^–7^. In this case, the value of parameter *F* used was 2.38, which was derived from eq 6. Solving eq 6 for (*x*,*y*) = (6,7) and *F* = 1.5 gives β = 7.1. Assuming that the value of β remains the same when the *T*_CutPNO_ ranges between 1.00 × 10^–6^ and 1.00 × 10^–7^, we solved eq 6 for *x* = 6 and *y* = 6.48 to find the respective *F* value. Application of this strategy on Mn SCO complexes was shown to correctly predict the ground spin state of the complexes.^64^ The second type of CPS extrapolation used is denoted as CPS2 and was performed using DLPNO-CCSD(T_1_) correlation energies obtained using two different *T*_CutPNO_ values, 1.00 × 10^–6^ and 1.00 × 10^–7^. In this case, the benchmarked value of parameter *F* 1.5 was used.

The CBS and CPS extrapolated correlation energies were calculated using the following formula:
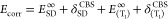
8where the additive correction terms δ_SD_^CBS^ and δ_(T_1_)_^CBS^ are derived from eq 4.

Canonical CCSD(T) calculations were performed for complex **1** starting from the quasi-restricted orbitals from the respective DLPNO-CC calculations, so that both canonical and DLPNO coupled cluster calculations have the same reference determinants.

As an indication of computational costs, we note that a DLPNO-CCSD(T_1_) calculation with NormalPNO settings running on 8 cores each with 25 GB memory for complex **1** (23 atoms) needs 6 h, for complex **4** (43 atoms), it needs 8.5 days, and for complex **12** (51 atoms), it needs 1.5 days.

## 3 Results and Discussion

### 3.1 Role of Perturbative Triple Approximations

We initially carried out DLPNO-CCSD(T_0_) calculations for complexes **1**–**12** with the default NormalPNO settings starting from a B3LYP reference determinant using the TZ/TZ basis sets. This protocol is similar to the one used by Phung et al.^74^ to compute the quintet–triplet adiabatic energy difference of complexes **6**–**12**. In that work, correlation consistent basis sets of valence triple-ζ size were used for Fe and O, and double-ζ basis sets were used on all remaining atoms. Our primary results along with the results by Feldt et al. for complexes **6**–**12** are given in Table 2, where the DLPNO-CC Δ*E* values are compared with CASPT2/CC results from the literature, which are used as reference values. Table 2 shows that DLPNO-CCSD(T_0_) calculations severely overstabilize the HS states with deviations from CASPT2/CC ranging from 17 to 4 kcal mol^–1^. Our results are therefore in complete agreement with those reported by Phung et al.^74^ Notably, sextet–doublet deviations for Fe(III) complexes are in all cases larger than sextet–quartet deviations by about 5 kcal mol^–1^. This kind of performance is clearly not useful for demanding applications.

**Table 2:** Spin-State Splittings Δ*E*, kcal mol^–1^, Obtained from DLPNO-CCSD(T) Calculations and Deviations ΔΔ*E* from the CASPT2/CC Benchmark^a^

		DLPNO-CCSD(T_0_)			DLPNO-CCSD(T_1_)		CASPT2/CC
		Δ*E*	Δ*E*^74^	ΔΔ*E*	Δ*E*	ΔΔ*E*	Δ*E*
1	^6^HS–^4^IS	–13.6		–9.0	–11.7	–7.0	–4.6^40^
	^6^HS–^2^LS	–20.9		–14.3	–15.6	–9.0	–6.6^40^
2	^6^HS–^4^IS	–19.2		–9.6	–17.4	–7.8	–9.6^40^
	^6^HS–^2^LS	–5.6		–14.0	–0.8	–9.2	8.5^40^
3	^6^HS–^4^IS	–18.6		–8.9	–16.7	–7.0	–9.7^40^
	^6^HS–^2^LS	–28.7		–14.6	–24.8	–10.7	–14.1^40^
4	^6^HS–^4^IS	–20.4		–8.9	–18.6	–7.1	–11.5^40^
	^6^HS–^2^LS	–9.4		–13.4	–5.7	–9.8	4.0^40^
5	^6^HS–^2^LS	–12.0		–17.1	–8.0	–13.1	5.1^41^
6	^5^HS–^3^LS	–5.0	–7.3	–5.4	–3.6	–4.0	0.4^74^
7	^5^HS–^3^LS	11.3	9.1	–5.8	15.0	–2.1	17.1^74^
8	^5^HS–^3^LS	25.2	25.7	–4.4	28.5	–1.1	29.6^74^
9	^5^HS–^3^LS	1.9	1.4	–8.3	4.1	–6.2	10.2^74^
10	^5^HS–^3^LS	4.2	3.9	–6.3	6.1	–4.4	10.5^74^
11	^5^HS–^3^LS	0.1	1.6	–9.0	2.1	–7.0	9.1^74^
12	^5^HS–^3^LS	3.1	3.5	–8.8	5.2	–6.7	11.9^74^

a The DLPNO-CC calculations were performed with the TZ/TZ basis set combination, UKS B3LYP reference orbitals, and NormalPNO settings.

^6^HS*–*^2^LS errors are larger than ^6^HS–^4^IS errors with all methods used here, since ^6^HS and ^2^LS have the largest differences in the recovery of correlation energy. This implies that the observed differences stem from the failure of the method to retrieve a sufficiently large part of the correlation energy to correct the HF imbalance in the treatment of states of different spins, i.e., the systematic overstabilization of HS states by HF. In the following, we investigate the possible sources of error in order to define a local coupled cluster protocol that represents an optimal compromise between computational cost and accuracy.

Initially, we examine deviations that originate from the approximations applied for the calculation of perturbative triple contributions. Within the DLPNO-CC framework, perturbative triple corrections can be computed using either the semicanonical triple corrections, denoted as (T_0_),^53^ or the more expensive improved triple corrections, denoted as (T_1_).^55^ In Table 2, the DLPNO-CCSD(T_0_) and (T_1_) computed spin-state splittings for complexes **1**–**12** are compared. Using (T_1_) proves crucial because in all cases it leads to closer agreement with CASPT2/CC, reducing the ΔΔ*E* values by 2 up to 5 kcal mol^–1^ relative to (T_0_). This is in agreement with a recent observation by Feldt et al.^75^ The main difference between the (T_0_) and (T_1_) treatments is that (T_0_) neglects nondiagonal terms of the Fock matrix; therefore, (T_1_) recovers a larger part of the triple correlation energy.^53,55^ Even though the (T_0_) approach has been reported to be sufficient for many cases,^54,56^ this is not in general the case for open-shell systems. The use of (T_0_) significantly compromises spin-state splittings of transition metal complexes because it retrieves a smaller percent of the canonical (T) correlation energy in the LS states than in the HS states, leading to overstabilization of the latter.^57,59,64^ This difference presumably relates to the existence of low-lying electronic states that render the nondiagonal terms of the Fock matrix non-negligible.^57^ Importantly, although the use of (T_1_) is clearly important, it is not sufficient to reconcile DLPNO-CCSD(T) with CASPT/CC, and therefore, additional sources of error must be identified.

### 3.2 Extrapolation to the PNO Space Limit

In order to evaluate the error that stems from the DLPNO approximation itself, we investigated the dependence of the percentage of the recovered canonical CCSD(T) correlation energy on the applied thresholds for the representative complex **1**. The DLPNO-CCSD(T_1_) and canonical CCSD(T) correlation energies were based on the same reference determinant for each system. Specifically, the quasi-restricted orbitals that were generated for the respective DLPNO-CCSD(T_1_) calculations were used as the reference determinant for the respective CCSD(T) calculations. All calculations for the DLPNO error investigation were performed using the TZ/TZ basis sets. In Table 3, the ^6^HS, ^4^IS, and ^2^LS state correlation energy components calculated using different DLPNO-CCSD(T_1_) settings are compared to the canonical CCSD(T) values. In addition, the respective correlation energy contributions to the sextet–quartet and sextet–doublet spin-state splittings are compared in Table 3.

**Table 3:** Correlation Energy Contributions (a.u.) and Spin-State Splittings Δ*E* (kcal mol^–1^) of Complex **1**, [Fe^III^L_2_OH], Calculated Using Different DLPNO-CCSD(T_1_) Settings Compared to Canonical CCSD(T) Results, Obtained Using the Same Reference Determinant

		normalPNO			tightPNO			
		*T*_CutPNO_ 3.33 × 10^–7^	CPS1	CPS2	*T*_CutPNO_ 1.00 × 10^–7^	CPS1	CPS2	canonical CCSD(T)
^6^HS	SD	–2.84652	–2.84100	–2.84159	–2.83909	–2.83521	–2.83585	–2.83908
	(T_1_)	–0.14232	–0.14727	–0.14702	–0.14429	–0.14736	–0.14715	–0.14630
^4^IS	SD	–2.91695	–2.91591	–2.91747	–2.91204	–2.91020	–2.91171	–2.91554
	(T_1_)	–0.16296	–0.16825	–0.16764	–0.16487	–0.16831	–0.16777	–0.16686
^2^LS	SD	–2.98831	–2.99113	–2.99323	–2.98621	–2.98644	–2.98836	–2.99278
	(T_1_)	–0.18630	–0.19138	–0.19164	–0.18869	–0.19143	–0.19175	–0.19047
Δ*E*								
^6^HS–^4^IS	SD	44.20	47.01	47.61	45.78	47.05	47.60	47.98
	(T_1_)	12.95	13.17	12.94	12.91	13.15	12.94	12.90
^6^HS–^2^LS	SD	88.97	94.21	95.16	92.32	94.90	95.70	96.45
	(T_1_)	27.60	27.68	28.00	27.86	27.66	27.99	27.72

**Figure 2:**
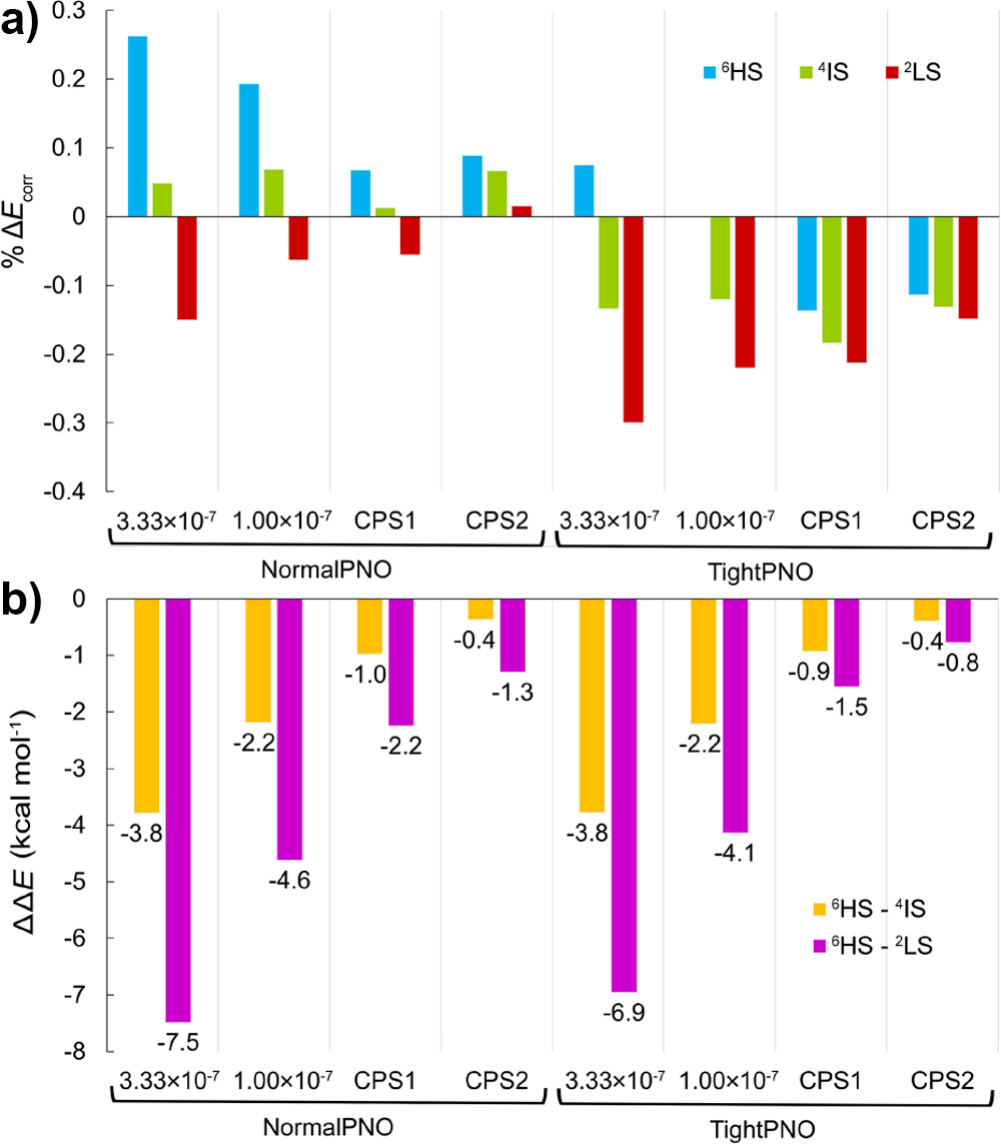
(a) *E*_corr_(DLPNO-CCSD) error relative to canonical CCSD in the calculated absolute energies of the ^6^HS shown in blue, the ^4^IS shown in green, and the ^2^LS shown in red, calculated using different DLPNO thresholds; left to right: default NormalPNO settings, NormalPNO settings with *T*_CutPNO_ = 1.00 × 10^–7^, CPS1 extrapolation from NormalPNO settings with *T*_CutPNO_ = 1.00 × 10^–6^ and *T*_CutPNO_ = 3.33 × 10^–7^, CPS2 extrapolation from NormalPNO settings with *T*_CutPNO_ = 1.00 × 10^–6^ and *T*_CutPNO_ = 1.00 × 10^–7^, TightPNO settings with *T*_CutPNO_ = 3.33 × 10^–7^, default TightPNO settings, CPS1 extrapolation from TightPNO settings with *T*_CutPNO_ = 1.00 × 10^–6^ and *T*_CutPNO_ = 3.33 × 10^–7^, and CPS2 extrapolation from TightPNO settings with *T*_CutPNO_ = 1.00 × 10^–6^ and *T*_CutPNO_ = 1.00 × 10^–7^; (b) DLPNO-CCSD correlation energy contributions to the adiabatic spin-state relative energies errors with respect to CCSD, yellow ^6^HS–^4^IS and purple ^6^HS–^2^LS, calculated with the above settings.

We begin by analyzing the single and double correlation energy contributions. In the DLPNO framework, the single and double correlation energy, *E*_corr_(DLPNO-CCSD), is the sum of the CCSD correlation energy from the “strong pairs” and the local-MP2 correlation energy from the “weak pairs”. The % errors of the DLPNO-CCSD correlation energies with respect to canonical CCSD, estimated using % Δ*E*_corr_ = [*E*_corr_(DLPNO-CCSD) – *E*_corr_(CCSD)]/*E*_corr_(CCSD)] × 100, for each structure in the respective spin state (^6^HS in blue, ^4^IS in green, and ^2^LS in red) and for each set of settings (x axis) are plotted in Figure 2a. When default NormalPNO settings (Table 1) are used, the DLPNO-CCSD overestimates the single and double correlation energy contributions of the ^6^HS state by 0.26% and those of the ^4^IS state by 0.05%, whereas it underestimates them in the ^2^LS state by 0.15%. The net overestimation of the correlation energy is the result of the local-MP2 overshooting the correlation energy. What is more interesting in these results is the relative error between the three spin states. As previously remarked,^75^ the problem in DLPNO stems from different percentages of *E*_corr_(CCSD) recovered for the different spin states. This is not only observed here for NormalPNO settings but also for the default TightPNO calculations. The bars in Figure 2b represent the resulting error in the spin-state splittings, i.e., ΔΔ*E* = Δ*E*^DLPNO-CCSD^ – Δ*E*^CCSD^. Apparently, the differences in the *E*_corr_ recovery percentage among the different spin states result in large deviations of 3.8 and 7.5 kcal mol^–1^ in the SD contributions to the relative energies favoring the HS state. The inadequate performance of the NormalPNO settings for metal complexes has been recognized in previous studies.^57,62^ Looking at the results obtained using the default TightPNO settings (Table 1), the *E*_corr_(CCSD) of the ^6^HS state is reproduced exactly, which is a result of error cancellation due to the local-MP2 overshooting, whereas those of the ^4^IS and ^2^LS states are underestimated by 0.12 and 0.22%, respectively. In this case, the recovered correlation energies are smaller because fewer pairs are characterized as “weak” when TightPNO settings are used (due to tighter *T*_CutPairs_ threshold, Table 1); hence, the local-MP2 contribution is diminished. Errors in the relative energies are reduced by 50% relative to the default NormalPNO settings, but the method is not yet converged.

In order to evaluate the impact of the *T*_CutPNO_ threshold relative to the other parameters (given in Table 1), we also carried out DLPNO-CCSD(T_1_) calculations using the NormalPNO and TightPNO settings but changing only the *T*_CutPNO_ value to 1.00 × 10^–7^ and 3.33 × 10^–7^, respectively. As expected, tighter *T*_CutPNO_ thresholds lead to SD values closer to canonical CCSD. Interestingly, NormalPNO and TightPNO settings with the same *T*_CutPNO_ value give very similar ΔΔΕ values. Hence, the main source of error in the spin-state splittings in the present case is the PNO space truncation error.

Application of the CPS2 extrapolation using TightPNO settings achieves nearly equal *E*_corr_(CCSD) recovery among the three spin states, leading to ΔΔ*Ε* values of only −0.4 and −0.8 kcal mol^–1^. Extrapolation to the CPS limit significantly decreases the differences in *E*_corr_(CCSD) recovery among the different spin states, and this is observed in all methods of two-point extrapolation used here. Notably, CPS2 extrapolation using NormalPNO settings is almost equally successful, introducing only 0.5 kcal mol^–1^ error in the sextet–doublet ΔΔ*Ε* value, which can be attributed to the pair truncation error. However, in some cases, calculations with a *T*_CutPNO_ value of 1.00 × 10^–7^ could become prohibitively expensive. Therefore, we also evaluate the performance of the alternative CPS1 extrapolation, which is based on the assumption that constant β (from eq 6) does not change between *T*_CutPNO_ values 1.00 × 10^–6^ and 1.00 × 10^–7^. Using the NormalPNO settings, the two-point CPS1 extrapolation overstabilizes the ^6^HS state by 1.0 and 2.2 kcal mol^–1^ relative to the ^4^IS and ^2^LS states, respectively. Therefore, the error of the optimal CPS2 extrapolation, which involves using TightPNO settings and changing only the *T*_CutPNO_ value, is increased by less than 1.5 kcal mol^–1^ albeit with significantly reduced computational effort.

Interestingly, changing the *T*_CutPNO_ threshold does not have a significant effect on relative energies attributed to triple excitations, as shown in Table 3. Even though increasing the *T*_CutPNO_ threshold enhances the recovery of the canonical perturbative triple correlation energy, *E*_corr_[(T)] by the *E*_corr_[DLPNO-(T_1_)], the errors of the DLPNO-(T_1_) correlation energies with respect to canonical triples (T), estimated using % Δ*E*_corr_ = [*E*_corr_(DLPNO-(T_1_)) – *E*_corr_(T)]/*E*_corr_(T)] × 100, remain similar for the three states (Figure S1a). Therefore, errors in relative energies do not show a specific dependence on PNO thresholds and are smaller than 0.3 kcal mol^–1^ (Figure S1b).

**Figure 3:**
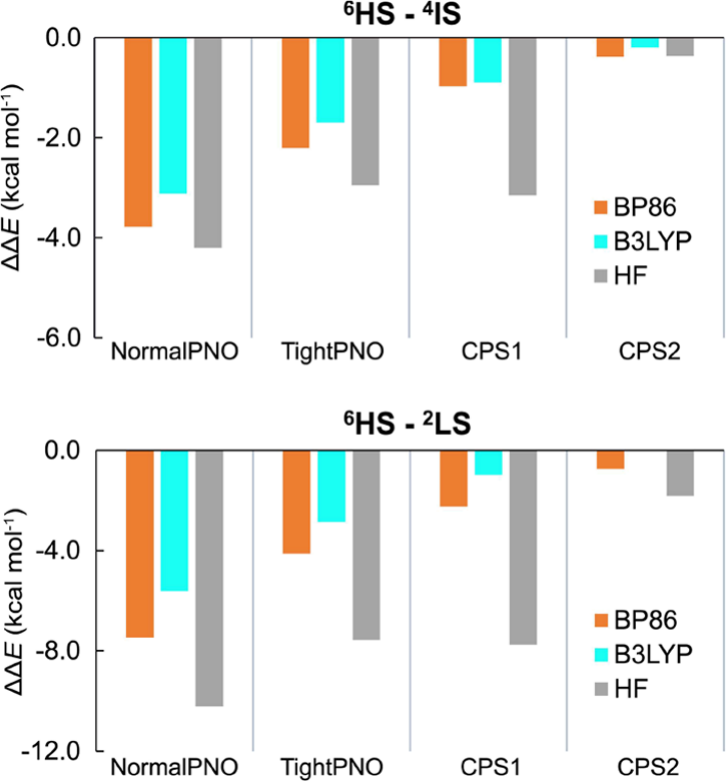
*E*_corr_(DLPNO-CCSD) contributions to the adiabatic spin-state relative energy errors with respect to *E*_corr_(CCSD) with BP86, B3LYP, and HF reference orbitals.

Furthermore, the impact of the choice of reference determinant on the DLPNO error is assessed. To this end, we compared the percentage of *E*_corr_(CCSD) recovery by DLPNO-CCSD calculations with BP86, B3LYP, and HF reference determinants. It is noted that Kohn–Sham orbitals are considered to provide better reference determinants for CCSD(T) than HF^41,73,89^ because they already include effects of orbital relaxation due to electron correlation. The *E*_corr_(CCSD) values were calculated based on the respective reference determinant. The errors ΔΔ*E* of the *E*_corr_(DLPNO-CCSD) correlation contributions with respect to canonical CCSD are compared in Figure 3. Detailed results are presented in Tables S1 and S2. It can be seen that using HF reference orbitals leads to the largest DLPNO errors, while DFT reference orbitals perform similarly. The crucial observation here is that CPS2 extrapolation with TightPNO settings minimizes the dependence of ΔΔ*E* on the reference determinant.

### 3.3 Comparison of DLPNO-CCSD(T) with CASPT2/CC

**Figure 4:**
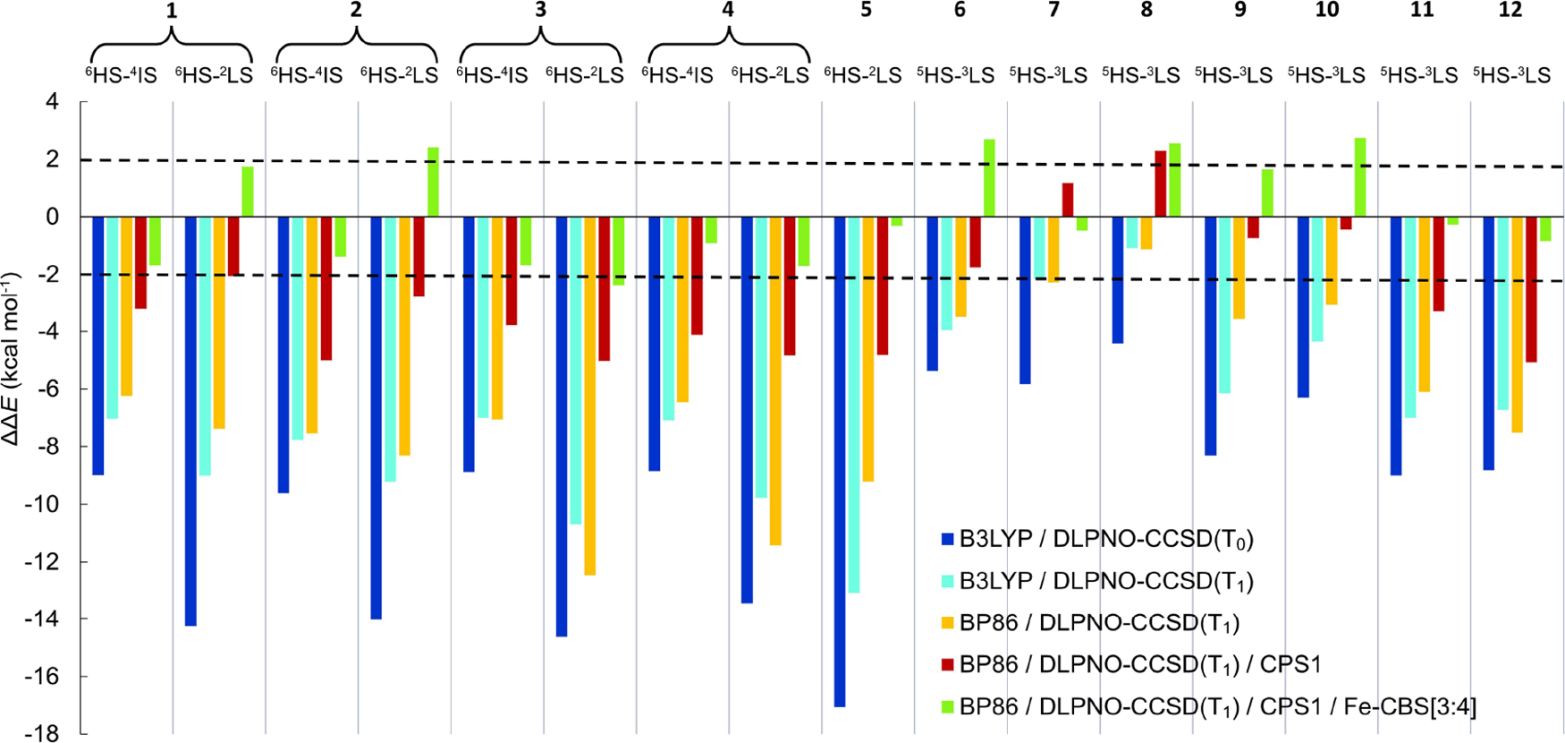
Error of the DLPNO-CCSD(T_1_) spin-state splittings Δ*E* with respect to CASPT2/CC reference values.

Having investigated the principal sources of error on the small complex **1**, we proceed to the application of the DLPNO-CCSD(T_1_)/CPS1 protocol on the benchmark set of complexes **1**–**12**. The Δ*E* values obtained from DLPNO-CCSD(T_1_) calculations with *T*_CutPNO_ 1.00 × 10^–6^ and 3.33 × 10^–7^ using the TZ/TZ basis sets and based on BP86 determinants are given in Table 4. Since the reference CASPT2/CC values are considered of CBS-limit quality, DLPNO-CCSD(T_1_) calculations with *T*_CutPNO_ 3.33 × 10^–7^ using the QZ/TZ basis sets were performed, in order to obtain the correction term δ^CBS^ (eq 4) to account for the basis set incompleteness error on Fe. These calculations were performed using NormalPNO settings (Table 1) and changing only the *T*_CutPNO_ value in order to perform the CPS1 extrapolation. Detailed numerical values for the correlation energy components of the individual structures are given in Tables S2–S13. The mean signed error (MSE) and mean unsigned error (MUE) of each method against the CASPT2/CC benchmark are also given in Table 3. In the bar chart of Figure 4, the performances of selected DLPNO-CCSD(T) protocols for spin-state energy differences are compared. The canonical CCSD(T) ΔΔ*E* values are expected to be between 0 and +2 kcal mol^–1^, since the CASPT2/CC method was reported to favor HS states relative to CCSD(T) by up to 2 kcal mol^–1^.^40^

**Table 4:** Spin-State Splittings (kcal mol^–1^) with DLPNO-CCSD(T_1_) Using BP86 Reference Orbitals and NormalPNO Initial Settings

		DLPNO-CCSD(T_1_)						CASPT2/CC
	Δ*E*	*T*_CutPNO_ 1.00 × 10^–6^ TZ/TZ	*T*_CutPNO_ 3.33 × 10^–7^ TZ/TZ	CPS1 TZ/TZ	*T*_CutPNO_ 3.33 × 10^–7^ QZ/TZ	δ^CBS^	CPS1 CBS[3:4]	
1	^6^HS–^4^IS	–13.1	–10.9	–7.8	–10.1	1.5	–6.3	–4.6
	^6^HS–^2^LS	–17.8	–14.0	–8.7	–12.1	3.8	–4.9	–6.6
2	^6^HS–^4^IS	–19.0	–17.2	–14.6	–15.1	3.6	–11.0	–9.6
	^6^HS–^2^LS	–3.8	0.2	5.7	3.0	5.2	10.9	8.5
3	^6^HS–^4^IS	–19.1	–16.7	–13.5	–15.6	2.1	–11.3	–9.7
	^6^HS–^2^LS	–31.9	–26.6	–19.1	–25.2	2.7	–16.5	–14.1
4	^6^HS–^4^IS	–19.7	–18.0	–15.6	–16.2	3.2	–12.4	–11.5
	^6^HS–^2^LS	–12.2	–7.4	–0.8	–5.7	3.1	2.3	4.0
5	^6^HS–^2^LS	–7.3	–4.1	0.3	–1.7	4.5	4.8	5.1
6	^5^HS–^3^LS	–4.4	–3.1	–1.4	–0.6	4.5	3.1	0.4
7	^5^HS–^3^LS	12.3	14.8	18.3	13.8	–1.7	16.6	17.1
8	^5^HS–^3^LS	26.0	28.5	31.9	28.5	0.3	32.6	29.6
9	^5^HS–^3^LS	4.6	6.6	9.5	7.9	2.4	11.8	10.2
10	^5^HS–^3^LS	5.5	7.4	10.1	9.2	3.2	13.2	10.5
11	^5^HS–^3^LS	1.0	3.0	5.8	4.7	3.0	8.82	9.1
12	^5^HS–^3^LS	2.6	4.4	6.8	6.8	4.2	11.1	11.9
	MSE	–9.2	–6.5	–2.7	–4.9		0.1	
	MUE	9.2	6.5	3.2	4.9		1.6	

As shown also in Table 2, the DLPNO-CCSD(T_0_) calculations based on the B3LYP reference determinant severely overstabilize HS states in all cases with an MSE of −9.9 kcal mol^–1^. The light blue bars represent ΔΔ*E* values calculated with (T_1_) instead of (T_0_) perturbative triple correction treatment. The MSE is reduced to −7.0 kcal mol^–1^ when the more expensive (T_1_) approximation is employed. In the present set of iron compounds, DLPNO-CCSD(T_1_) calculations starting with BP86 orbitals give slightly better accuracy on average than with the B3LYP reference, with MSE values of −7.0 and −6.5 kcal mol^–1^, respectively. However, it is important to point out that the BP86 reference does not provide better DLPNO-CCSD(T) results than the B3LYP reference for all complexes, since **3**, **4**, and **12** show the reverse behavior.

The CPS extrapolation of the DLPNO-CCSD(T_1_) correlation energies leads to further stabilization of the lower spin states, diminishing the ΔΔ*E* values, represented by the red bars, up to 6 kcal mol^–1^, with negligible additional computational cost. The MSE after the CPS extrapolation is −2.7 kcal mol^–1^. Addition of the correction δ^CBS^ to include the effect of basis set extrapolation in all cases leads to further improvement. Hence, the differences of the DLPNO-CCSD(T_1_) calculated values with both CPS and Fe CBS[3:4] extrapolations applied simultaneously (green bars) are in the range between −2.4 and 2.7 kcal mol^–1^ with respect to the CASPT2/CC reference, with an MSE of 0.1 kcal mol^–1^. We should note here that the reported^40^ CASPT2/CC tendency to overstabilize the HS states with respect to canonical CCSD(T) shows that the presented DLPNO-CC protocol might also on average reproduce this bias, which is expected to be corrected using the CPS2 extrapolation protocol. Most importantly, the same effects are observed for all complexes of the set, which shows that the effects are systematic and implies that the DLPNO-CCSD(T_1_)/CPS1/CBS[3:4] protocol is transferable to other Fe^III^ and Fe^IV^–oxo complexes outside our benchmark set.

## 4 Conclusions

In this work, we investigated the sources of error that cause the systematic overstabilization of the HS states by the DLPNO-CCSD(T) method for spin-state splittings of iron complexes that was reported in recent studies.^73−75^ A reliable protocol that reproduces the reference CASPT2/CC spin-state splittings with minimal additional computational effort is proposed. We have demonstrated that the semicanonical perturbative triple (T_0_) correction is inappropriate for describing spin-state splittings in these systems and the use of the improved perturbative triple (T_1_) corrections is required. We strongly suggest that the (T_1_) approach should be considered as the only acceptable option for applications of DLPNO-CCSD(T) to problems of spin-state energetics. Overstabilization of HS states stems also from the differences in the percentage of the canonical CCSD correlation energy recovered by DLPNO between different spin states. On the choice of the thresholds that adjust the size of the correlation space, the domain error was found to be significantly more important than the pair error, based on the spin-state splitting deviations from canonical CCSD values that depend mostly on the *T*_CutPNO_ threshold. Two-point extrapolation to the CPS limit using DLPNO-CCSD(T_1_) correlation energies obtained with *T*_CutPNO_ values 1.00 × 10^–6^ and 1.00 × 10^–7^, which have been suggested as optimal by Bistoni and co-workers,^77^ greatly improves the accuracy of the method. The CPS extrapolation reduces the deviation between the DLPNO-CCSD and the canonical CCSD correlation energies, reduces the dependence of results on the reference determinant, and, most importantly, eliminates the relative errors on the correlation energy recovery between different spin states. Attempting to define a protocol that maintains the benefits of the CPS extrapolation but at reduced cost to make the approach applicable to the complete set of complexes, we converged to a protocol that involves CPS extrapolation with *T*_CutPNO_ values 1.00 × 10^–6^ and 3.33 × 10^–7^. This can push the accuracy limits of DLPNO-CCSD(T) with very small additional computational cost compared to a standard NormalPNO calculation, which is encouraging for cases where TightPNO calculations are not affordable, as well as for lower-level parts in multilevel approaches.^90−92^ Combination of CPS extrapolation with Fe-centered two-point extrapolation to the CBS limit further improves the accuracy of the computed values. The presented combined DLPNO-CCSD(T) protocol correctly identifies the ground spin states in all iron complexes studied herein and yields spin-state splittings that faithfully reproduce the CASPT2/CC benchmark values within the stated accuracy of the latter. This is especially significant because the straightforward and easily implemented protocol presented in this work promises to deliver reliable spin-state energetics even for larger and more complex systems that would be computationally too demanding for multireference approaches.

## Supporting Information Available

*E*_corr_[DLPNO-(T_1_)] error relative to canonical triples (T) in the calculated absolute energies and correlation energy contributions to the adiabatic spin-state relative energies for complex **1** (Figure S1); detailed results of CCSD(T) calculations for complex **1** (Table S1); detailed results of DLPNO-CCSD(T) calculations for all complexes (Tables S2–S14); and Cartesian coordinates of all complexes (PDF)

## Notes

The original ORCA output files are provided as an open-access data set hosted by the Open Research Data Repository of the Max Planck Society at https://doi.org/10.17617/3.XWIA1R.
