# Supplementary material for: Reconciling Local Coupled Cluster with Multireference Approaches for Transition Metal Spin-State Energetics
Source: J Chem Theory Comput. 2022 May 18;18(6):3538–48. doi: 10.1021/acs.jctc.2c00265 (PMC9202354; doi:10.1021/acs.jctc.2c00265)
Supplement: Supplementary file 1 — ct2c00265_si_001.pdf [file ct2c00265_si_001.pdf]

Supporting Information

for

Reconciling local coupled cluster and multireference approaches for transition metal spin-state energetics

Maria Drosou,*[a] Christiana A. Mitsopoulou,[a] Dimitrios A. Pantazis*[b]

[a] Inorganic Chemistry Laboratory, National and Kapodistrian University of Athens, Panepistimiopolis, Zografou 15771, Greece

[b] Max-Planck-Institut für Kohlenforschung, Kaiser-Wilhelm-Platz 1, 45470 Mülheim an der Ruhr, Germany

* Email: mdrosou@chem.uoa.gr, dimitrios.pantazis@kofo.mpg.de

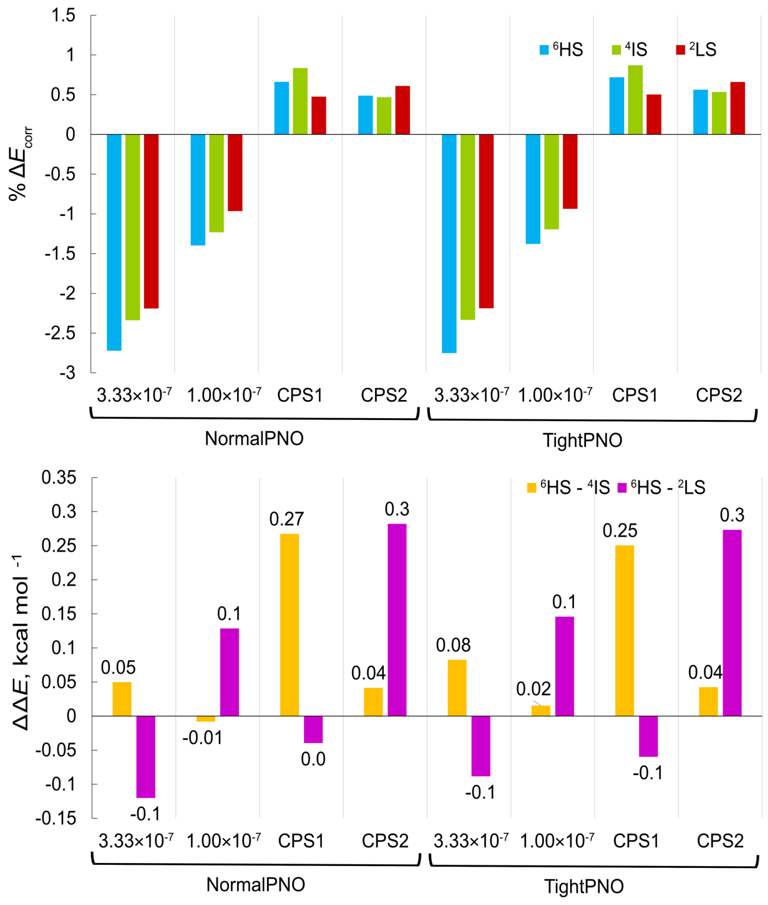

Figure S1. a) E_{corr}[DLPNO-(T_1)] error relative to canonical triples (T) in the calculated absolute energies of the ^{6}HS shown in blue, the ^{4}IS shown in green and the ^{2}LS shown in red, calculated using different DLPNO thresholds; left to right: default NormalPNO settings, NormalPNO settings with T_{CutPNO} = 1.00×10^{-7}, CPS1 extrapolation using NormalPNO settings with T_{CutPNO} = 1.00×10^{-6} and 3.33×10^{-7}, CPS2 extrapolation using NormalPNO settings with T_{CutPNO} = 1.00×10^{-6} and 1.00×10^{-7}, TightPNO settings with T_{CutPNO} = 3.33×10^{-7}, default TightPNO settings, CPS1 extrapolation using TightPNO settings with T_{CutPNO} = 1.00×10^{-6} and 3.33×10^{-7}, CPS2 extrapolation using TightPNO settings with T_{CutPNO} =1.00×10^{-6} and 1.00×10^{-7}, **b)** E_{corr}[DLPNO-(T_1)] energy contributions to the adiabatic spin state relative energies errors with respect to canonical CCSD(T) triples, yellow ^{6}HS–^{4}IS and purple ^{6}HS–^{2}LS, calculated with the above settings.

Table S1. Detailed results of CCSD(T) calculations for complex **1**. Absolute energies for each state are given in a.u. and spin-splittings Δ*E* in kcal mol^{-1}.

	BP86 reference / CCSD(T)			
	HF	CCSD	(T)	*E* (HF + E_{corr})
^{6}HS	-1803.194598	-2.839083	-0.146305	-1806.179986
^{4}IS	-1803.086207	-2.915542	-0.166861	-1806.168610
Δ*E*				
^{6}HS-^{4}IS	-68.02	47.98	12.90	-7.14
^{2}LS	-1802.986546	-2.992783	-0.190473	-1806.169802
Δ*E*				
^{6}HS-^{2}LS	-130.56	96.45	27.72	-6.39
	B3LYP reference / CCSD(T)			
	HF	CCSD	(T)	*E* (HF + E_{corr})
^{6}HS	-1803.290305	-2.752476	-0.141037	-1806.183818
^{4}IS	-1803.191671	-2.820748	-0.158742	-1806.171161
Δ*E*				
^{6}HS-^{4}IS	-61.89	42.84	11.11	-7.94
^{2}LS	-1803.117744	-2.875347	-0.177385	-1806.170476
Δ*E*				
^{6}HS-^{2}LS	-108.28	77.10	22.81	-8.37
	HF reference / CCSD(T)			
	HF	CCSD	(T)	*E* (HF + E_{corr})
^{6}HS	-1803.483809	-2.591736	-0.122356	-1806.197901
^{4}IS	-1803.386835	-2.662139	-0.134853	-1806.183827
Δ*E*				
^{6}HS-^{4}IS	-60.85	44.18	7.84	-8.83
^{2}LS	-1803.322189	-2.711734	-0.147585	-1806.181509
Δ*E*				
^{6}HS-^{2}LS	-101.42	75.30	15.83	-10.29

Table S2. Detailed results of DLPNO-CCSD(T) calculations for complex **1**. Absolute energies for each state are given in a.u. and spin-splittings Δ*E* in kcal mol^{-1}. The CCSD energy is the canonical CCSD correlation energy from the "strong pairs" and the LPM2 energy is the local-MP2 correlation energy from the "weak pairs". The total energy, *E* is defined as the sum of the HF, CCSD, MP2 and (T_1) energies.

BP86 reference / DLPNO-CCSD(T) / NormalPNO, T_{CutPNO} = 1.00×10^{-6} / TZ/TZ basis sets							
	BP86	HF	CCSD	LMP2	(T_0)	(T_1)	E (HF + E_{corr})
^{6}HS	-1808.230412	-1803.194598	-2.782283	-0.068221	-0.131373	-0.138749	-1806.183851
^{4}IS	-1808.249137	-1803.086207	-2.859209	-0.058495	-0.147688	-0.159133	-1806.163045
Δ*E*							
^{6}HS-^{4}IS	11.75	-68.02	48.27	-6.10	10.24	12.79	-13.06
^{2}LS	-1808.262732	-1802.986546	-2.940936	-0.045329	-0.163416	-0.182633	-1806.155444
Δ*E*							
^{6}HS-^{2}LS	20.28	-130.56	99.56	-14.37	20.11	27.54	-17.83
BP86 reference / DLPNO-CCSD(T) / NormalPNO, T_{CutPNO} = 3.33×10^{-7} / TZ/TZ basis sets							
	BP86	HF	CCSD	LMP2	(T_0)	(T_1)	E (HF + E_{corr})
^{6}HS	-1808.230412	-1803.194598	-2.806958	-0.039559	-0.134616	-0.142324	-1806.183439
^{4}IS	-1808.249137	-1803.086207	-2.882567	-0.034382	-0.150927	-0.162959	-1806.166115
Δ*E*							
^{6}HS-^{4}IS	11.75	-68.02	47.45	-3.25	10.24	12.95	-10.87
^{2}LS	-1808.262732	-1802.986546	-2.962968	-0.025337	-0.166241	-0.186301	-1806.161152
Δ*E*							
^{6}HS-^{2}LS	20.28	-130.56	97.90	-8.92	19.84	27.60	-13.99
BP86 reference / DLPNO-CCSD(T) / NormalPNO, T_{CutPNO} = 3.33×10^{-7} / QZ/TZ basis sets							
	BP86	HF	CCSD	LMP2	(T_0)	(T_1)	E (HF + E_{corr})
^{6}HS	-1808.277469	-1803.237822	-2.849701	-0.045268	-0.137990	-0.145815	-1806.278607
^{4}IS	-1808.296063	-1803.129005	-2.928342	-0.038491	-0.154522	-0.166707	-1806.262545
Δ*E*							
^{6}HS-^{4}IS	11.67	-68.28	49.35	-4.25	10.37	13.11	-10.08
^{2}LS	-1808.309016	-1803.028438	-3.011334	-0.028893	-0.170318	-0.190674	-1806.259339
Δ*E*							
^{6}HS-^{2}LS	19.80	-131.39	101.43	-10.28	20.29	28.15	-12.09
B3LYP reference / DLPNO-CCSD(T) / NormalPNO, T_{CutPNO} = 3.33×10^{-7} / TZ/TZ basis sets							
	B3LYP	HF	CCSD	LMP2	(T_0)	(T_1)	E (HF + E_{corr})
^{6}HS	-1807.629556	-1803.290304	-2.721528	-0.037074	-0.128563	-0.135547	-1806.184454
^{4}IS	-1807.629407	-1803.191671	-2.789598	-0.032304	-0.142202	-0.152300	-1806.165874
Δ*E*							
^{6}HS-^{4}IS	-0.09	-61.89	42.71	-2.99	8.56	10.51	-11.66
^{2}LS	-1807.624506	-1803.117744	-2.848097	-0.024419	-0.153955	-0.169314	-1806.159575
Δ*E*							
^{6}HS-^{2}LS	-3.17	-108.28	79.42	-7.94	15.93	21.19	-15.61
B3LYP reference / DLPNO-CCSD(T) / NormalPNO, T_{CutPNO} = 1.00×10^{-6} / TZ/TZ basis sets							

	B3LYP	HF	CCSD	LMP2	(T_0)	(T_1)	E (HF + E_{corr})
^{6}HS	-1807.629753	-1803.290304	-2.698334	-0.063301	-0.125465	-0.132139	-1806.184078
^{4}IS	-1807.624697	-1803.117744	-2.828220	-0.041985	-0.150611	-0.165009	-1806.152957
Δ*E*							
^{6}HS-^{4}IS	-3.17	-108.28	81.50	-13.38	15.78	20.63	-19.53
^{2}LS	-1807.629407	-1803.191671	-2.770067	-0.048445	-0.139209	-0.148818	-1806.159001
Δ*E*							
^{6}HS-^{2}LS	-0.09	-61.89	44.31	-6.16	8.43	10.23	-13.52
		HF reference / DLPNO-CCSD(T) / NormalPNO, T_{CutPNO} = 3.33×10^{-7} / TZ/TZ basis sets					
		HF	CCSD	LMP2	(T_0)	(T_1)	E (HF + E_{corr})
^{6}HS	-1803.490531	-1803.483809	-2.554376	-0.030621	-0.109379	-0.114271	-1806.183077
^{4}IS	-1803.396147	-1803.386835	-2.622331	-0.026377	-0.117608	-0.123816	-1806.159358
Δ*E*							
^{6}HS-^{4}IS	-59.23	-60.85	42.64	-2.66	5.16	5.99	-14.88
^{2}LS	-1803.342255	-1803.322189	-2.667702	-0.021022	-0.123260	-0.131141	-1806.142054
Δ*E*							
^{6}HS-^{2}LS	-93.04	-101.42	71.11	-6.02	8.71	10.59	-25.74
		HF reference / DLPNO-CCSD(T) / NormalPNO, T_{CutPNO} = 1.00×10^{-6} / TZ/TZ basis sets					
		HF	CCSD	LMP2	(T_0)	(T_1)	E (HF + E_{corr})
^{6}HS	-1803.490531	-1803.483809	-2.533934	-0.048645	-0.106391	-0.111010	-1806.177398
^{4}IS	-1803.396147	-1803.386835	-2.602094	-0.042989	-0.114162	-0.119914	-1806.151832
Δ*E*							
^{6}HS-^{4}IS	-59.23	-60.85	42.77	-3.55	4.88	5.59	-16.04
^{2}LS	-1803.342255	-1803.322189	-2.649156	-0.034327	-0.119526	-0.126677	-1806.132350
Δ*E*							
^{6}HS-^{2}LS	-93.04	-101.42	72.30	-8.98	8.24	9.83	-28.27

Table S3. Detailed results of DLPNO-CCSD(T) calculations for complex **2**. Absolute energies for each state are given in a.u. and spin-splittings Δ*E* in kcal mol^{-1}. The CCSD energy is the canonical CCSD correlation energy from the "strong pairs" and the LPM2 energy is the local-MP2 correlation energy from the "weak pairs". The total energy, *E* is defined as the sum of the HF, CCSD, MP2 and (T_1) energies.

BP86 reference / DLPNO-CCSD(T) / NormalPNO, T_{CutPNO} = 1.00×10^{-6} / TZ/TZ basis sets							
	BP86	HF	CCSD	LMP2	(T_0)	(T_1)	*E* (HF + E_{corr})
^{6}HS	-1864.852359	-1859.438373	-3.034391	-0.071708	-0.139895	-0.147355	-1862.691827
^{4}IS	-1864.862399	-1859.315276	-3.115774	-0.062677	-0.156438	-0.167813	-1862.661539
Δ*E*							
^{6}HS-^{4}IS	6.30	-77.24	51.07	-5.67	10.38	12.84	-19.01
^{2}LS	-1864.908655	-1859.246517	-3.199264	-0.049469	-0.172229	-0.190476	-1862.685726
Δ*E*							
^{6}HS-^{2}LS	35.33	-120.39	103.46	-13.96	20.29	27.06	-3.83
BP86 reference / DLPNO-CCSD(T) / NormalPNO, T_{CutPNO} = 3.33×10^{-7} / TZ/TZ basis sets							
	BP86	HF	CCSD	LMP2	(T_0)	(T_1)	*E* (HF + E_{corr})
^{6}HS	-1864.852359	-1859.438373	-3.059556	-0.043026	-0.143008	-0.150765	-1862.691719
^{4}IS	-1864.862399	-1859.315276	-3.140194	-0.037409	-0.159543	-0.171474	-1862.664353
Δ*E*							
^{6}HS-^{4}IS	6.30	-77.24	50.60	-3.52	10.38	13.00	-17.17
^{2}LS	-1864.908655	-1859.246517	-3.221962	-0.029339	-0.175127	-0.194143	-1862.691961
Δ*E*							
^{6}HS-^{2}LS	35.33	-120.39	101.91	-8.59	20.16	27.22	0.15
BP86 reference / DLPNO-CCSD(T) / NormalPNO, T_{CutPNO} = 3.33×10^{-7} / QZ/TZ basis sets							
	BP86	HF	CCSD	LMP2	(T_0)	(T_1)	*E* (HF + E_{corr})
^{6}HS	-1864.899319	-1859.481015	-3.103019	-0.049120	-0.146496	-0.154418	-1862.787572
^{4}IS	-1864.909718	-1859.357973	-3.187672	-0.042309	-0.163446	-0.175591	-1862.763545
Δ*E*							
^{6}HS-^{4}IS	6.53	-77.21	53.12	-4.27	10.64	13.29	-15.08
^{2}LS	-1864.955320	-1859.288648	-3.272243	-0.032833	-0.179322	-0.198674	-1862.792398
Δ*E*							
^{6}HS-^{2}LS	35.14	-120.71	106.19	-10.22	20.60	27.77	3.03

Table S4. Detailed results of DLPNO-CCSD(T) calculations for complex **3**. Absolute energies for each state are given in a.u. and spin-splittings Δ*E* in kcal mol^{-1}. The CCSD energy is the canonical CCSD correlation energy from the "strong pairs" and the LPM2 energy is the local-MP2 correlation energy from the "weak pairs". The total energy, *E* is defined as the sum of the HF, CCSD, MP2 and (T_1) energies.

	BP86 reference / DLPNO-CCSD(T) / NormalPNO, T_{CutPNO} = 1.00×10^{-6} / TZ/TZ basis sets						
	BP86	HF	CCSD	LMP2	(T_0)	(T_1)	*E* (HF + E_{corr})
^{6}HS	-2343.518824	-2334.931212	-4.911825	-0.134854	-0.267344	-0.285677	-2340.263568
^{4}IS	-2343.528704	-2334.817361	-4.986301	-0.122305	-0.284871	-0.307178	-2340.233146
Δ*E*							
^{6}HS-^{4}IS	6.20	-71.44	46.73	-7.87	11.00	13.49	-19.09
^{2}LS	-2343.531066	-2334.739692	-5.039522	-0.109288	-0.296330	-0.324181	-2340.212683
Δ*E*							
^{6}HS-^{2}LS	7.68	-120.18	80.13	-16.04	18.19	24.16	-31.93
	BP86 reference / DLPNO-CCSD(T) / NormalPNO, T_{CutPNO} = 3.33×10^{-7} / TZ/TZ basis sets						
	BP86	HF	CCSD	LMP2	(T_0)	(T_1)	*E* (HF + E_{corr})
^{6}HS	-2343.518824	-2334.931212	-4.956585	-0.085118	-0.274747	-0.294109	-2340.267024
^{4}IS	-2343.528704	-2334.817361	-5.028166	-0.079135	-0.292114	-0.315709	-2340.240372
Δ*E*							
^{6}HS-^{4}IS	6.20	-71.44	44.92	-3.75	10.90	13.55	-16.72
^{2}LS	-2343.531066	-2334.739692	-5.080949	-0.071101	-0.303462	-0.332963	-2340.224706
Δ*E*							
^{6}HS-^{2}LS	7.68	-120.18	78.04	-8.80	18.02	24.38	-26.55
	BP86 reference / DLPNO-CCSD(T) / NormalPNO, T_{CutPNO} = 3.33×10^{-7} / QZ/TZ basis sets						
	BP86	HF	CCSD	LMP2	(T_0)	(T_1)	*E* (HF + E_{corr})
^{6}HS	-2343.564812	-2334.973665	-4.997983	-0.091071	-0.277952	-0.297404	-2340.360123
^{4}IS	-2343.574422	-2334.859478	-5.072515	-0.084022	-0.295525	-0.319267	-2340.335282
Δ*E*							
^{6}HS-^{4}IS	6.03	-71.65	46.77	-4.42	11.03	13.72	-15.59
^{2}LS	-2343.576404	-2334.781244	-5.127203	-0.074776	-0.307096	-0.336696	-2340.319919
Δ*E*							
^{6}HS-^{2}LS	7.27	-120.75	81.09	-10.23	18.29	24.66	-25.23

Table S5. Detailed results of DLPNO-CCSD(T) calculations for complex **4**. Absolute energies for each state are given in a.u. and spin-splittings Δ*E* in kcal mol^{-1}. The CCSD energy is the canonical CCSD correlation energy from the "strong pairs" and the LPM2 energy is the local-MP2 correlation energy from the "weak pairs". The total energy, *E* is defined as the sum of the HF, CCSD, MP2 and (T_1) energies.

	BP86 reference / DLPNO-CCSD(T) / NormalPNO, T_{CutPNO} = 1.00×10^{-6} / TZ/TZ basis sets						
	BP86	HF	CCSD	LMP2	(T_0)	(T_1)	*E* (HF + E_{corr})
^{6}HS	-2400.142753	-2391.172743	-5.174237	-0.139357	-0.276790	-0.295317	-2396.781654
^{4}IS	-2400.151710	-2391.056608	-5.247327	-0.130545	-0.293492	-0.315846	-2396.750326
Δ*E*							
^{6}HS-^{4}IS	5.62	-72.88	45.86	-5.53	10.48	12.88	-19.66
^{2}LS	-2400.190289	-2391.005009	-5.306439	-0.117418	-0.305768	-0.333421	-2396.762287
Δ*E*							
^{6}HS-^{2}LS	29.83	-105.25	82.96	-13.77	18.18	23.91	-12.15
	BP86 reference / DLPNO-CCSD(T) / NormalPNO, T_{CutPNO} = 3.33×10^{-7} / TZ/TZ basis sets						
	BP86	HF	CCSD	LMP2	(T_0)	(T_1)	*E* (HF + E_{corr})
^{6}HS	-2400.142753	-2391.172743	-5.218104	-0.091392	-0.284115	-0.303652	-2396.785892
^{4}IS	-2400.151710	-2391.056608	-5.289170	-0.087082	-0.300782	-0.324393	-2396.757253
Δ*E*							
^{6}HS-^{4}IS	5.62	-72.88	44.59	-2.70	10.46	13.02	-17.97
^{2}LS	-2400.190289	-2391.005009	-5.348287	-0.078856	-0.312799	-0.341971	-2396.774122
Δ*E*							
^{6}HS-^{2}LS	29.83	-105.25	81.69	-7.87	18.00	24.05	-7.39
	BP86 reference / DLPNO-CCSD(T) / NormalPNO, T_{CutPNO} = 3.33×10^{-7} / QZ/TZ basis sets						
	BP86	HF	CCSD	LMP2	(T_0)	(T_1)	*E* (HF + E_{corr})
^{6}HS	-2400.188610	-2391.214619	-5.261029	-0.096979	-0.287477	-0.307130	-2396.879755
^{4}IS	-2400.197598	-2391.098330	-5.336291	-0.091353	-0.304294	-0.328033	-2396.854007
Δ*E*							
^{6}HS-^{4}IS	5.64	-72.97	47.23	-3.53	10.55	13.12	-16.16
^{2}LS	-2400.235682	-2391.046562	-5.396031	-0.081987	-0.316758	-0.346160	-2396.870740
Δ*E*							
^{6}HS-^{2}LS	29.54	-105.46	84.72	-9.41	18.37	24.49	-5.66

Table S6. Detailed results of DLPNO-CCSD(T) calculations for complex **5**. Absolute energies for each state are given in a.u. and spin-splittings Δ*E* in kcal mol^{-1}. The CCSD energy is the canonical CCSD correlation energy from the "strong pairs" and the LPM2 energy is the local-MP2 correlation energy from the "weak pairs". The total energy, *E* is defined as the sum of the HF, CCSD, MP2 and (T_1) energies.

	BP86 reference / DLPNO-CCSD(T) / NormalPNO, T_{CutPNO} = 1.00×10^{-6} / TZ/TZ basis sets						
	BP86	HF	CCSD	LMP2	(T_0)	(T_1)	*E* (HF + E_{corr})
^{6}HS	-2117.665836	-2110.584823	-4.074841	-0.108182	-0.188777	-0.199973	-2114.967818
^{2}LS	-2117.699218	-2110.407796	-4.228679	-0.079047	-0.219113	-0.240685	-2114.956207
Δ*E*							
^{6}HS-^{2}LS	20.95	-111.09	96.53	-18.28	19.04	25.55	-7.29
	BP86 reference / DLPNO-CCSD(T) / NormalPNO, T_{CutPNO} = 3.33×10^{-7} / TZ/TZ basis sets						
	BP86	HF	CCSD	LMP2	(T_0)	(T_1)	*E* (HF + E_{corr})
^{6}HS	-2117.665836	-2110.584823	-4.109403	-0.070314	-0.193780	-0.205496	-2114.970035
^{2}LS	-2117.699218	-2110.407796	-4.257115	-0.052882	-0.223266	-0.245693	-2114.963487
Δ*E*							
^{6}HS-^{2}LS	20.95	-111.09	92.69	-10.94	18.50	25.22	-4.11
	BP86 reference / DLPNO-CCSD(T) / NormalPNO, T_{CutPNO} = 3.33×10^{-7} / QZ/TZ basis sets						
	BP86	HF	CCSD	LMP2	(T_0)	(T_1)	*E* (HF + E_{corr})
^{6}HS	-2117.711045	-2110.626768	-4.151485	-0.076145	-0.197058	-0.208869	-2115.063267
^{2}LS	-2117.744184	-2110.448848	-4.305520	-0.056464	-0.227078	-0.249699	-2115.060531
Δ*E*							
^{6}HS-^{2}LS	20.80	-111.65	96.66	-12.35	18.84	25.62	-1.72

Table S7. Detailed results of DLPNO-CCSD(T) calculations for complex **6**. Absolute energies for each state are given in a.u. and spin-splittings ΔE in kcal mol^{-1}. The CCSD energy is the canonical CCSD correlation energy from the "strong pairs" and the LPM2 energy is the local-MP2 correlation energy from the "weak pairs". The total energy, E is defined as the sum of the HF, CCSD, MP2 and (T_1) energies.

	BP86 reference / DLPNO-CCSD(T) / NormalPNO, T_{CutPNO} = 1.00×10^{-6} / TZ/TZ basis sets						
	BP86	HF	CCSD	LMP2	(T_0)	(T_1)	E (HF + E_{corr})
^{5}HS	-1636.06943	-1632.062194	-2.091008	-0.043564	-0.102216	-0.111116	-1634.307882
^{3}LS	-1636.08668	-1631.992427	-2.149512	-0.033024	-0.114377	-0.12599	-1634.300953
ΔE							
^{5}HS-^{3}LS	10.83	-43.78	36.71	-6.61	7.63	9.33	-4.35
	BP86 reference / DLPNO-CCSD(T) / NormalPNO, T_{CutPNO} = 3.33×10^{-7} / TZ/TZ basis sets						
	BP86	HF	CCSD	LMP2	(T_0)	(T_1)	E (HF + E_{corr})
^{5}HS	-1636.069431	-1632.062194	-2.1080599	-0.026933	-0.10365	-0.112701	-1634.309888
^{3}LS	-1636.086684	-1631.992427	-2.1642833	-0.020621	-0.115633	-0.127626	-1634.304957
ΔE							
^{5}HS-^{3}LS	10.83	-43.78	35.28	-3.96	7.52	9.37	-3.09
	BP86 reference / DLPNO-CCSD(T) / NormalPNO, T_{CutPNO} = 3.33×10^{-7} / QZ/TZ basis sets						
	BP86	HF	CCSD	LMP2	(T_0)	(T_1)	E (HF + E_{corr})
^{5}HS	-1636.116	-1632.104188	-2.153933	-0.032073	-0.107395	-0.116414	-1634.4066
^{3}LS	-1636.133	-1632.034055	-2.215201	-0.024512	-0.119779	-0.131858	-1634.4056
ΔE							
^{5}HS-^{3}LS	10.77	-44.01	38.45	-4.74	7.77	9.69	-0.62

Table S8. Detailed results of DLPNO-CCSD(T) calculations for complex **7**. Absolute energies for each state are given in a.u. and spin-splittings Δ*E* in kcal mol^{-1}. The CCSD energy is the canonical CCSD correlation energy from the "strong pairs" and the LPM2 energy is the local-MP2 correlation energy from the "weak pairs". The total energy, *E* is defined as the sum of the HF, CCSD, MP2 and (T_1) energies.

BP86 reference / DLPNO-CCSD(T) / NormalPNO, T_{CutPNO} = 1.00×10^{-6} / TZ/TZ basis sets							
	BP86	HF	CCSD	LMP2	(T_0)	(T_1)	*E* (HF + E_{corr})
^{5}HS	-2490.100406	-2480.416791	-5.665567	-0.131618	-0.317821	-0.338296	-2486.552272
^{3}LS	-2490.129425	-2480.413407	-5.696420	-0.112132	-0.323324	-0.349938	-2486.571897
Δ*E*							
^{5}HS-^{3}LS	18.21	-2.12	19.36	-12.23	3.45	7.31	12.31
BP86 reference / DLPNO-CCSD(T) / NormalPNO, T_{CutPNO} = 3.33×10^{-7} / TZ/TZ basis sets							
	BP86	HF	CCSD	LMP2	(T_0)	(T_1)	*E* (HF + E_{corr})
^{5}HS	-2490.100406	-2480.416791	-5.708960	-0.087325	-0.324816	-0.346183	-2486.559259
^{3}LS	-2490.129425	-2480.413407	-5.734648	-0.077508	-0.329550	-0.357307	-2486.582871
Δ*E*							
^{5}HS-^{3}LS	18.21	-2.12	16.12	-6.16	2.97	6.98	14.82
BP86 reference / DLPNO-CCSD(T) / NormalPNO, T_{CutPNO} = 3.33×10^{-7} / QZ/TZ basis sets							
	BP86	HF	CCSD	LMP2	(T_0)	(T_1)	*E* (HF + E_{corr})
^{5}HS	-2490.144887	-2480.456766	-5.751953	-0.094202	-0.328685	-0.350166	-2486.653087
^{3}LS	-2490.173685	-2480.453024	-5.780606	-0.080710	-0.332973	-0.360715	-2486.675056
Δ*E*							
^{5}HS-^{3}LS	18.07	-2.35	17.98	-8.47	2.69	6.62	13.79

Table S9. Detailed results of DLPNO-CCSD(T) calculations for complex **8**. Absolute energies for each state are given in a.u. and spin-splittings Δ*E* in kcal mol^{-1}. The CCSD energy is the canonical CCSD correlation energy from the "strong pairs" and the LPM2 energy is the local-MP2 correlation energy from the "weak pairs". The total energy, *E* is defined as the sum of the HF, CCSD, MP2 and (T_1) energies.

	BP86 reference / DLPNO-CCSD(T) / NormalPNO, T_{CutPNO} = 1.00×10^{-6} / TZ/TZ basis sets						
	BP86	HF	CCSD	LMP2	(T_0)	(T_1)	*E* (HF + E_{corr})
^{5}HS	-2623.026855	-2612.482087	-6.209643	-0.139568	-0.343201	-0.364894	-2619.196192
^{3}LS	-2623.067780	-2612.472301	-6.261460	-0.124434	-0.351808	-0.379442	-2619.237637
Δ*E*							
^{5}HS-^{3}LS	25.68	-6.14	32.52	-9.50	5.40	9.13	26.01
	BP86 reference / DLPNO-CCSD(T) / NormalPNO, T_{CutPNO} = 3.33×10^{-7} / TZ/TZ basis sets						
	BP86	HF	CCSD	LMP2	(T_0)	(T_1)	*E* (HF + E_{corr})
^{5}HS	-2623.026855	-2612.482087	-6.254726	-0.093980	-0.350336	-0.372939	-2619.203733
^{3}LS	-2623.067780	-2612.472301	-6.301150	-0.088457	-0.358377	-0.387206	-2619.249114
Δ*E*							
^{5}HS-^{3}LS	25.68	-6.14	29.13	-3.47	5.05	8.95	28.48
	BP86 reference / DLPNO-CCSD(T) / NormalPNO, T_{CutPNO} = 3.33×10^{-7} / QZ/TZ basis sets						
	BP86	HF	CCSD	LMP2	(T_0)	(T_1)	*E* (HF + E_{corr})
^{5}HS	-2623.071353	-2612.522051	-6.297836	-0.100348	-0.354098	-0.376783	-2619.297019
^{3}LS	-2623.111860	-2612.511727	-6.347704	-0.092113	-0.362076	-0.390902	-2619.342447
Δ*E*							
^{5}HS-^{3}LS	25.42	-6.48	31.29	-5.17	5.01	8.86	28.51

Table S10. Detailed results of DLPNO-CCSD(T) calculations for complex **9**. Absolute energies for each state are given in a.u. and spin-splittings Δ*E* in kcal mol^{-1}. The CCSD energy is the canonical CCSD correlation energy from the "strong pairs" and the LPM2 energy is the local-MP2 correlation energy from the "weak pairs". The total energy, *E* is defined as the sum of the HF, CCSD, MP2 and (T_1) energies.

BP86 reference / DLPNO-CCSD(T) / NormalPNO, T_{CutPNO} = 1.00×10^{-6} / TZ/TZ basis sets							
	BP86	HF	CCSD	LMP2	(T_0)	(T_1)	E (HF + E_{corr})
^{5}HS	-2258.343419	-2249.896444	-4.757678	-0.109474	-0.235031	-0.250644	-2255.014240
^{3}LS	-2258.365229	-2249.828218	-4.825412	-0.097233	-0.250760	-0.270670	-2255.021533
Δ*E*							
^{5}HS-^{3}LS	13.69	-42.81	42.50	-7.68	9.87	12.57	4.58
BP86 reference / DLPNO-CCSD(T) / NormalPNO, T_{CutPNO} = 3.33×10^{-7} / TZ/TZ basis sets							
	BP86	HF	CCSD	LMP2	(T_0)	(T_1)	E (HF + E_{corr})
^{5}HS	-2258.343419	-2249.896444	-4.788930	-0.077632	-0.239428	-0.255475	-2255.018480
^{3}LS	-2258.365229	-2249.828218	-4.854258	-0.071034	-0.254896	-0.275528	-2255.029038
Δ*E*							
^{5}HS-^{3}LS	13.69	-42.81	40.99	-4.14	9.71	12.58	6.63
BP86 reference / DLPNO-CCSD(T) / NormalPNO, T_{CutPNO} = 3.33×10^{-7} / QZ/TZ basis sets							
	BP86	HF	CCSD	LMP2	(T_0)	(T_1)	E (HF + E_{corr})
^{5}HS	-2258.388377	-2249.937619	-4.834591	-0.083716	-0.243100	-0.259091	-2255.115018
^{3}LS	-2258.410149	-2249.869039	-4.903969	-0.075176	-0.258807	-0.279449	-2255.127633
Δ*E*							
^{5}HS-^{3}LS	13.66	-43.03	43.54	-5.36	9.86	12.77	7.92

Table S11. Detailed results of DLPNO-CCSD(T) calculations for complex **10**. Absolute energies for each state are given in a.u. and spin-splittings Δ*E* in kcal mol^{-1}. The CCSD energy is the canonical CCSD correlation energy from the "strong pairs" and the LPM2 energy is the local-MP2 correlation energy from the "weak pairs". The total energy, *E* is defined as the sum of the HF, CCSD, MP2 and (T_1) energies.

BP86 reference / DLPNO-CCSD(T) / NormalPNO, T_{CutPNO} = 1.00×10^{-6} / TZ/TZ basis sets							
	BP86	HF	CCSD	LMP2	(T_0)	(T_1)	*E* (HF + E_{corr})
^{5}HS	-2253.573256	-2245.317769	-4.848151	-0.107341	-0.254216	-0.271485	-2250.544746
^{3}LS	-2253.599508	-2245.245991	-4.922293	-0.096740	-0.267273	-0.288509	-2250.553532
Δ*E*							
^{5}HS-^{3}LS	16.47	-45.04	46.52	-6.65	8.19	10.68	5.51
BP86 reference / DLPNO-CCSD(T) / NormalPNO, T_{CutPNO} = 3.33×10^{-7} / TZ/TZ basis sets							
	BP86	HF	CCSD	LMP2	(T_0)	(T_1)	*E* (HF + E_{corr})
^{5}HS	-2253.573256	-2245.317769	-4.882081	-0.072856	-0.259112	-0.276883	-2250.549590
^{3}LS	-2253.599508	-2245.245991	-4.953752	-0.067753	-0.271919	-0.293917	-2250.561413
Δ*E*							
^{5}HS-^{3}LS	16.47	-45.04	44.97	-3.20	8.04	10.69	7.42
BP86 reference / DLPNO-CCSD(T) / NormalPNO, T_{CutPNO} = 3.33×10^{-7} / QZ/TZ basis sets							
	BP86	HF	CCSD	LMP2	(T_0)	(T_1)	*E* (HF + E_{corr})
^{5}HS	-2253.617879	-2245.358395	-4.925553	-0.078387	-0.262642	-0.280353	-2250.642688
^{3}LS	-2253.644052	-2245.286169	-5.001696	-0.071660	-0.275726	-0.297746	-2250.657270
Δ*E*							
^{5}HS-^{3}LS	16.42	-45.32	47.78	-4.22	8.21	10.91	9.15

Table S12. Detailed results of DLPNO-CCSD(T) calculations for complex **11**. Absolute energies for each state are given in a.u. and spin-splittings Δ*E* in kcal mol^{-1}. The CCSD energy is the canonical CCSD correlation energy from the "strong pairs" and the LPM2 energy is the local-MP2 correlation energy from the "weak pairs". The total energy, *E* is defined as the sum of the HF, CCSD, MP2 and (T_1) energies.

	BP86 reference / DLPNO-CCSD(T) / NormalPNO, T_{CutPNO} = 1.00×10^{-6} / TZ/TZ basis sets						
	BP86	HF	CCSD	LMP2	(T_0)	(T_1)	*E* (HF + E_{corr})
^{5}HS	-2253.577034	-2245.329004	-4.842053	-0.108752	-0.253530	-0.270345	-2250.550154
^{3}LS	-2253.597095	-2245.246312	-4.919674	-0.097663	-0.267082	-0.288100	-2250.551749
Δ*E*							
^{5}HS-^{3}LS	12.59	-51.89	48.71	-6.96	8.50	11.14	1.00
	BP86 reference / DLPNO-CCSD(T) / NormalPNO, T_{CutPNO} = 3.33×10^{-7} / TZ/TZ basis sets						
	BP86	HF	CCSD	LMP2	(T_0)	(T_1)	*E* (HF + E_{corr})
^{5}HS	-2253.577034	-2245.329004	-4.875964	-0.073939	-0.258440	-0.275749	-2250.554657
^{3}LS	-2253.597095	-2245.246312	-4.951006	-0.068605	-0.271748	-0.293529	-2250.559451
Δ*E*							
^{5}HS-^{3}LS	12.59	-51.89	47.09	-3.35	8.35	11.16	3.01
	BP86 reference / DLPNO-CCSD(T) / NormalPNO, T_{CutPNO} = 3.33×10^{-7} / QZ/TZ basis sets						
	BP86	HF	CCSD	LMP2	(T_0)	(T_1)	*E* (HF + E_{corr})
^{5}HS	-2253.621659	-2245.369563	-4.919558	-0.079145	-0.261925	-0.279180	-2250.647445
^{3}LS	-2253.641612	-2245.286470	-4.998832	-0.072308	-0.275482	-0.297270	-2250.654880
Δ*E*							
^{5}HS-^{3}LS	12.52	-52.14	49.75	-4.29	8.51	11.35	4.67

Table S13. Detailed results of DLPNO-CCSD(T) calculations for complex **12**. Absolute energies for each state are given in a.u. and spin-splittings Δ*E* in kcal mol^{-1}. The CCSD energy is the canonical CCSD correlation energy from the "strong pairs" and the LPM2 energy is the local-MP2 correlation energy from the "weak pairs". The total energy, *E* is defined as the sum of the HF, CCSD, MP2 and (T_1) energies.

	BP86 reference / DLPNO-CCSD(T) / NormalPNO, T_{CutPNO} = 1.00×10^{-6} / TZ/TZ basis sets						
	BP86	HF	CCSD	LMP2	(T_0)	(T_1)	*E* (HF + E_{corr})
^{5}HS	-2517.025348	-2507.149735	-5.427537	-0.122251	-0.306069	-0.328670	-2513.028193
^{3}LS	-2517.057649	-2507.061791	-5.513668	-0.110232	-0.319628	-0.346665	-2513.032356
Δ*E*							
^{5}HS-^{3}LS	20.27	-55.19	54.05	-7.54	8.51	11.29	2.61
	BP86 reference / DLPNO-CCSD(T) / NormalPNO, T_{CutPNO} = 3.33×10^{-7} / TZ/TZ basis sets						
	BP86	HF	CCSD	LMP2	(T_0)	(T_1)	*E* (HF + E_{corr})
^{5}HS	-2517.025348	-2507.149735	-5.462597	-0.086257	-0.311575	-0.334835	-2513.033424
^{3}LS	-2517.057649	-2507.061791	-5.545659	-0.080072	-0.324854	-0.352885	-2513.040407
Δ*E*							
^{5}HS-^{3}LS	20.27	-55.19	52.12	-3.88	8.33	11.33	4.38
	BP86 reference / DLPNO-CCSD(T) / NormalPNO, T_{CutPNO} = 3.33×10^{-7} / QZ/TZ basis sets						
	BP86	HF	CCSD	LMP2	(T_0)	(T_1)	*E* (HF + E_{corr})
^{5}HS	-2517.070612	-2507.191229	-5.506987	-0.091691	-0.315067	-0.338282	-2513.128190
^{3}LS	-2517.102989	-2507.103027	-5.595285	-0.084271	-0.328432	-0.356376	-2513.138958
Δ*E*							
^{5}HS-^{3}LS	20.32	-55.35	55.41	-4.66	8.39	11.35	6.76

Table S14. Spin contamination values of the UKS BP86 and B3LYP reference determinants used for the construction of the quasi-restricted orbitals for the DLPNO-CC calculations.

Structure		BP86	B3LYP
**1**	^{6}HS	0.0081	0.0112
	^{4}IS	0.0249	0.0300
	^{2}LS	0.0291	0.0574
**2**	^{6}HS	0.0074	0.0097
	^{4}IS	0.0295	0.0328
	^{2}LS	0.0159	0.0196
**3**	^{6}HS	0.0074	0.0108
	^{4}IS	0.0300	0.0359
	^{2}LS	0.0462	0.1024
**4**	^{6}HS	0.0088	0.0110
	^{4}IS	0.0335	0.0366
	^{2}LS	0.0221	0.0195
**5**	^{6}HS	0.0084	0.0111
	^{2}LS	0.0161	0.0301
**6**	^{5}HS	0.0265	0.0550
	^{3}LS	0.0099	0.0156
**7**	^{5}HS	0.0339	0.0661
	^{3}LS	0.0174	0.0269
**8**	^{5}HS	0.0338	0.0655
	^{3}LS	0.0177	0.0322
**9**	^{5}HS	0.0317	0.0598
	^{3}LS	0.0183	0.0288
**10**	^{5}HS	0.0342	0.0638
	^{3}LS	0.0158	0.0223
**11**	^{5}HS	0.0314	0.0570
	^{3}LS	0.0153	0.0234
**12**	^{5}HS	0.0279	0.0563
	^{3}LS	0.0158	0.0199

Cartesian Coordinates of the Studied Iron Complexes

Coordinates of complexes **1**-**4** were taken from reference 1, of complex **5** from reference 2 and of complexes **6**-**12** from reference 3.

Complex **1**, [Fe^{IIIL}2OH]

^{6}HS

Fe	0.019465000	0.298652000	0.000000000
N	-1.387794000	-0.216025000	1.445197000
N	-1.387794000	-0.216025000	-1.445197000
N	1.405569000	-0.308043000	-1.433258000
N	1.405569000	-0.308043000	1.433258000
C	-1.221100000	-0.246714000	2.757314000
C	0.016999000	-0.262301000	3.415492000
C	1.245173000	-0.341358000	2.749115000
C	-1.221100000	-0.246714000	-2.757314000
C	0.016999000	-0.262301000	-3.415492000
C	1.245173000	-0.341358000	-2.749115000
H	-2.117708000	-0.283850000	3.392896000
H	0.019549000	-0.292315000	4.503098000
H	2.140728000	-0.458784000	3.376540000
H	2.382386000	-0.439820000	1.165658000
H	-2.374967000	-0.247985000	1.185034000
H	-2.374967000	-0.247985000	-1.185034000
H	-2.117708000	-0.283850000	-3.392896000
H	0.019549000	-0.292315000	-4.503098000
H	2.140728000	-0.458784000	-3.376540000
H	2.382386000	-0.439820000	-1.165658000
O	0.043617000	2.150887000	0.000000000
H	0.886764000	2.632950000	0.000000000

^{4}IS

Fe	0.022114000	0.116409000	0.000000000
N	-1.346391000	-0.199940000	1.352932000
N	-1.346391000	-0.199940000	-1.352932000
N	1.371721000	-0.271401000	-1.350745000
N	1.371721000	-0.271401000	1.350745000
C	-1.209944000	-0.228765000	2.666754000
C	0.014373000	-0.235716000	3.346604000
C	1.232657000	-0.315458000	2.667573000
C	-1.209944000	-0.228765000	-2.666754000
C	0.014373000	-0.235716000	-3.346604000
C	1.232657000	-0.315458000	-2.667573000
H	-2.125322000	-0.272315000	3.271493000
H	0.013035000	-0.269398000	4.433258000
H	2.142402000	-0.441707000	3.269305000
H	2.343989000	-0.389655000	1.057335000
H	-2.325655000	-0.218497000	1.060403000
H	-2.325655000	-0.218497000	-1.060403000
H	-2.125322000	-0.272315000	-3.271493000
H	0.013035000	-0.269398000	-4.433258000
H	2.142402000	-0.441707000	-3.269305000
H	2.343989000	-0.389655000	-1.057335000
O	0.028545000	2.008137000	0.000000000
H	0.895128000	2.449259000	0.000000000

^{2}LS

Fe	0.016601000	0.015992000	0.000000000
N	-1.336854000	-0.181847000	1.324012000
N	-1.336854000	-0.181847000	-1.324012000
N	1.359063000	-0.243506000	-1.318747000
N	1.359063000	-0.243506000	1.318747000
C	-1.212628000	-0.208256000	2.645216000
C	0.013922000	-0.212742000	3.319448000
C	1.234970000	-0.274196000	2.644357000
C	-1.212628000	-0.208256000	-2.645216000
C	0.013922000	-0.212742000	-3.319448000

C	1.234970000	-0.274196000	-2.644357000
H	-2.129395000	-0.259784000	3.245328000
H	0.014052000	-0.245181000	4.406835000
H	2.149169000	-0.387300000	3.239794000
H	2.335879000	-0.339399000	1.024636000
H	-2.317151000	-0.188489000	1.028444000
H	-2.317151000	-0.188489000	-1.028444000
H	-2.129395000	-0.259784000	-3.245328000
H	0.014052000	-0.245181000	-4.406835000
H	2.149169000	-0.387300000	-3.239794000
H	2.335879000	-0.339399000	-1.024636000
O	-0.002374000	1.834197000	0.000000000
H	0.931234000	2.119314000	0.000000000

Complex **2**, [Fe^{III}L2(NH_3)(OH)]

^{6}HS

Fe	-0.015388000	-0.157829000	0.000000000
N	1.465683000	0.064479000	1.599500000
N	-1.469355000	0.060691000	1.519140000
N	-1.469355000	0.060691000	-1.519140000
N	1.465683000	0.064479000	-1.599500000
C	-0.050920000	0.062521000	3.502147000
C	-0.050920000	0.062521000	-3.502147000
C	1.218833000	0.030447000	2.896665000
C	-1.281985000	0.060414000	2.831099000
C	-1.281985000	0.060414000	-2.831099000
C	1.218833000	0.030447000	-2.896665000
H	-0.080946000	0.049865000	4.590517000
H	-0.080946000	0.049865000	-4.590517000
H	-2.460746000	0.009280000	1.278455000
H	-2.173875000	0.043436000	3.475920000
H	2.073618000	-0.025640000	3.589138000
H	2.464802000	-0.017015000	1.399505000
H	2.464802000	-0.017015000	-1.399505000
H	2.073618000	-0.025640000	-3.589138000
H	-2.173875000	0.043436000	-3.475920000
H	-2.460746000	0.009280000	-1.278455000
O	0.130124000	-2.025240000	0.000000000
H	0.954999000	-2.536401000	0.000000000
N	0.100448000	1.977283000	0.000000000
H	0.597064000	2.307462000	-0.831289000
H	0.597064000	2.307462000	0.831289000
H	-0.834820000	2.392064000	0.000000000

^{4}IS

Fe	-0.008478000	-0.084836000	0.000000000
N	1.399478000	0.012917000	1.433779000
N	-1.408040000	0.052368000	1.381097000
N	-1.408040000	0.052368000	-1.381097000
N	1.399478000	0.012917000	-1.433779000
C	-0.035873000	0.033087000	3.380892000
C	-0.035873000	0.033087000	-3.380892000
C	1.213456000	0.017338000	2.739552000
C	-1.256525000	0.029280000	2.695613000
C	-1.256525000	0.029280000	-2.695613000
C	1.213456000	0.017338000	-2.739552000
H	-0.056382000	0.026539000	4.468510000
H	-0.056382000	0.026539000	-4.468510000
H	-2.387657000	-0.013635000	1.096728000
H	-2.167763000	0.004512000	3.309481000
H	2.102117000	0.004221000	3.387055000
H	2.386313000	-0.040751000	1.172485000
H	2.386313000	-0.040751000	-1.172485000
H	2.102117000	0.004221000	-3.387055000
H	-2.167763000	0.004512000	-3.309481000
H	-2.387657000	-0.013635000	-1.096728000
O	0.059619000	-2.020319000	0.000000000
H	0.881202000	-2.539313000	0.000000000
N	0.103585000	2.050477000	0.000000000

H	0.583552000	2.418837000	-0.826789000
H	0.583552000	2.418837000	0.826789000
H	-0.841570000	2.446324000	0.000000000

^{2}LS

Fe	-0.013452000	-0.076184000	0.000000000
N	1.316078000	0.312589000	1.341597000
N	-1.371407000	-0.335288000	1.347050000
N	-1.371407000	-0.335288000	-1.347050000
N	1.316078000	0.312589000	-1.341597000
C	-0.025364000	-0.010795000	3.328115000
C	-0.025364000	-0.010795000	-3.328115000
C	1.169505000	0.284598000	2.661999000
C	-1.221833000	-0.318101000	2.661427000
C	-1.221833000	-0.318101000	-2.661427000
C	1.169505000	0.284598000	-2.661999000
H	-0.024385000	-0.011427000	4.416201000
H	-0.024385000	-0.011427000	-4.416201000
H	-2.301427000	-0.648292000	1.057577000
H	-2.096830000	-0.559350000	3.279527000
H	2.049826000	0.513485000	3.277247000
H	2.273491000	0.537438000	1.057463000
H	2.273491000	0.537438000	-1.057463000
H	2.049826000	0.513485000	-3.277247000
H	-2.096830000	-0.559350000	-3.279527000
H	-2.301427000	-0.648292000	-1.057577000
O	0.339667000	-1.888710000	0.000000000
H	1.310551000	-1.982775000	0.000000000
N	-0.522762000	2.000526000	0.000000000
H	-0.132307000	2.448560000	-0.832056000
H	-0.132307000	2.448560000	0.832056000
H	-1.536171000	2.135605000	0.000000000

Complex **3**, [Fe^{III}P(OH)]

^{6}HS

Fe	0.000000000	0.000000000	0.000000000
O	0.000000000	0.000000000	1.843077042
H	0.000000000	-0.850667372	2.314483663
N	-1.441280000	-1.447370806	-0.515419001
N	-1.442801000	1.447926865	-0.500122384
N	1.441280000	-1.447370806	-0.515419001
N	1.442801000	1.447926865	-0.500122384
C	2.803900000	1.247499373	-0.529953459
C	1.245181000	2.808973705	-0.544016084
C	2.519801000	3.487504434	-0.597929912
C	3.485488000	2.520199775	-0.587788719
C	3.432641000	0.001193917	-0.537296589
C	2.804041000	-1.244707801	-0.548216546
C	3.485135000	-2.515341011	-0.633803066
C	2.520114000	-3.483662392	-0.661059049
C	1.245095000	-2.808635894	-0.589313297
C	0.000000000	-3.439386595	-0.611048049
C	-1.245095000	-2.808635894	-0.589313297
C	-2.520114000	-3.483662392	-0.661059049
C	-3.485135000	-2.515341011	-0.633803066
C	-2.804041000	-1.244707801	-0.548216546
C	-3.432641000	0.001193917	-0.537296589
C	-2.803900000	1.247499373	-0.529953459
C	-3.485488000	2.520199775	-0.587788719
C	-2.519801000	3.487504434	-0.597929912
C	-1.245181000	2.808973705	-0.544016084
C	0.000000000	3.439347930	-0.553738887
H	0.000000000	4.529428175	-0.589933207
H	2.649489000	4.565645208	-0.644767257
H	4.564227000	2.647610169	-0.626121466
H	4.522987000	0.001110758	-0.567360505
H	4.563777000	-2.641237145	-0.680171254
H	2.651243000	-4.560305326	-0.733108251
H	0.000000000	-4.528360400	-0.673003118
H	-2.651243000	-4.560305326	-0.733108251

H	-4.563777000	-2.641237145	-0.680171254
H	-4.522987000	0.001110758	-0.567360505
H	-4.564227000	2.647610169	-0.626121466
H	-2.649489000	4.565645208	-0.644767257

^{4}IS

Fe	0.000000000	0.000000000	0.000000000
O	0.000000000	0.000000000	1.875852190
H	0.000000000	-0.882178911	2.288387850
N	-1.414498000	-1.411379866	-0.313065957
N	-1.414342000	1.421538283	-0.262639817
N	1.414498000	-1.411379866	-0.313065957
N	1.414342000	1.421538283	-0.262639817
C	2.780686000	1.234568311	-0.264294436
C	1.228883000	2.787502590	-0.267250381
C	2.498639000	3.468662399	-0.277570894
C	3.463131000	2.503414906	-0.273310585
C	3.424829000	0.005511648	-0.276100994
C	2.782607000	-1.223609344	-0.314657094
C	3.464031000	-2.489945670	-0.387438243
C	2.499530000	-3.454996483	-0.434876206
C	1.229527000	-2.777211489	-0.386819183
C	0.000000000	-3.420885760	-0.415117595
C	-1.229527000	-2.777211489	-0.386819183
C	-2.499530000	-3.454996483	-0.434876206
C	-3.464031000	-2.489945670	-0.387438243
C	-2.782607000	-1.223609344	-0.314657094
C	-3.424829000	0.005511648	-0.276100994
C	-2.780686000	1.234568311	-0.264294436
C	-3.463131000	2.503414906	-0.273310585
C	-2.498639000	3.468662399	-0.277570894
C	-1.228883000	2.787502590	-0.267250381
C	0.000000000	3.431754190	-0.264770597
H	0.000000000	4.521683669	-0.265978742
H	2.620222000	4.548514464	-0.287880757
H	4.543085000	2.624032374	-0.281160202
H	4.514801000	0.006253610	-0.277049461
H	4.543907000	-2.610076137	-0.407609479
H	2.622378000	-4.532793797	-0.500948613
H	0.000000000	-4.509298747	-0.473650122
H	-2.622378000	-4.532793797	-0.500948613
H	-4.543907000	-2.610076137	-0.407609479
H	-4.514801000	0.006253610	-0.277049461
H	-4.543085000	2.624032374	-0.281160202
H	-2.620222000	4.548514464	-0.287880757

^{2}LS

Fe	0.000000000	0.000000000	0.000000000
O	0.000000000	0.000000000	1.788339141
H	0.000000000	-0.935072826	2.077526214
N	-1.397995000	-1.409842812	-0.211948251
N	-1.399273000	1.413715582	-0.184554799
N	1.397995000	-1.409842812	-0.211948251
N	1.399273000	1.413715582	-0.184554799
C	2.768043000	1.229816903	-0.114593216
C	1.225065000	2.784461646	-0.245523126
C	2.496329000	3.462236638	-0.233696713
C	3.454406000	2.496252158	-0.143956971
C	3.407164000	0.001629729	-0.067882355
C	2.767871000	-1.226495270	-0.139222208
C	3.454020000	-2.490360650	-0.205982577
C	2.497192000	-3.455703774	-0.324524082
C	1.225646000	-2.781058785	-0.315723354
C	0.000000000	-3.425013310	-0.374751289
C	-1.225646000	-2.781058785	-0.315723354
C	-2.497192000	-3.455703774	-0.324524082
C	-3.454020000	-2.490360650	-0.205982577
C	-2.767871000	-1.226495270	-0.139222208
C	-3.407164000	0.001629729	-0.067882355
C	-2.768043000	1.229816903	-0.114593216

C	-3.454406000	2.496252158	-0.143956971
C	-2.496329000	3.462236638	-0.233696713
C	-1.225065000	2.784461646	-0.245523126
C	0.000000000	3.429949610	-0.285512930
H	0.000000000	4.519175012	-0.328313006
H	2.621715000	4.540296699	-0.287144211
H	4.534302000	2.613698713	-0.108883518
H	4.496179000	0.000699663	-0.015087706
H	4.534160000	-2.607402642	-0.177195611
H	2.624826000	-4.531331492	-0.411180505
H	0.000000000	-4.512316036	-0.453039873
H	-2.624826000	-4.531331492	-0.411180505
H	-4.534160000	-2.607402642	-0.177195611
H	-4.496179000	0.000699663	-0.015087706
H	-4.534302000	2.613698713	-0.108883518
H	-2.621715000	4.540296699	-0.287144211

Complex **4**, $[Fe^{III}P(OH)(NH_3)]$

^{6}HS

Fe	0.000000000	0.000000000	0.000000000
O	0.000000000	0.000000000	1.879476558
H	0.000000000	0.827456645	2.389409200
N	0.000000000	0.127318495	-2.092852543
H	0.000000000	-0.798515341	-2.529953711
H	0.824619000	0.629207798	-2.435075983
H	-0.824619000	0.629207798	-2.435075983
N	1.478564000	1.516372374	-0.137959267
N	1.474379000	-1.452138672	-0.257263769
N	-1.478564000	1.516372374	-0.137959267
N	-1.474379000	-1.452138672	-0.257263769
C	-2.827980000	-1.230909084	-0.224335027
C	-1.262094000	-2.805330608	-0.308067354
C	-2.545435000	-3.480119102	-0.314483243
C	-3.510443000	-2.509518072	-0.261832232
C	-3.443798000	0.030623027	-0.162628738
C	-2.830677000	1.295414431	-0.116416720
C	-3.513132000	2.573285251	-0.036491937
C	-2.546511000	3.543223341	-0.005307629
C	-1.263637000	2.867502643	-0.065650418
C	0.000000000	3.484207376	-0.040233372
C	1.263637000	2.867502643	-0.065650418
C	2.546511000	3.543223341	-0.005307629
C	3.513132000	2.573285251	-0.036491937
C	2.830677000	1.295414431	-0.116416720
C	3.443798000	0.030623027	-0.162628738
C	2.827980000	-1.230909084	-0.224335027
C	3.510443000	-2.509518072	-0.261832232
C	2.545435000	-3.480119102	-0.314483243
C	1.262094000	-2.805330608	-0.308067354
C	0.000000000	-3.423333409	-0.337868283
H	0.000000000	-4.514023060	-0.374382955
H	-2.686820000	-4.557634993	-0.347255135
H	-4.589120000	-2.644533924	-0.245025177
H	-4.534980000	0.027690652	-0.138960996
H	-4.591560000	2.707753852	-0.003817325
H	-2.686398000	4.619679646	0.057483200
H	0.000000000	4.573934556	0.021790965
H	2.686398000	4.619679646	0.057483200
H	4.591560000	2.707753852	-0.003817325
H	4.534980000	0.027690652	-0.138960996
H	4.589120000	-2.644533924	-0.245025177
H	2.686820000	-4.557634993	-0.347255135

^{4}IS

Fe	0.000000000	0.000000000	0.000000000
O	0.000000000	0.000000000	1.917472154
H	0.000000000	0.851235671	2.388755313
N	0.000000000	0.083384355	-2.095063500
H	0.000000000	-0.853565741	-2.509898685
H	0.823127000	0.571896407	-2.461354613

H	-0.823127000	0.571896407	-2.461354613
N	1.442675000	1.455612124	-0.074092314
N	1.433336000	-1.426673217	-0.127408534
N	-1.442675000	1.455612124	-0.074092314
N	-1.433336000	-1.426673217	-0.127408534
C	-2.794520000	-1.225054724	-0.096054125
C	-1.239600000	-2.787429147	-0.140465990
C	-2.514960000	-3.465775412	-0.126159377
C	-3.478243000	-2.497592166	-0.097208571
C	-3.432097000	0.013675609	-0.066962231
C	-2.802134000	1.257500600	-0.055570708
C	-3.483965000	2.531271921	-0.023713156
C	-2.517185000	3.497169211	-0.020625568
C	-1.242754000	2.815318748	-0.049944912
C	0.000000000	3.449002016	-0.044047257
C	1.242754000	2.815318748	-0.049944912
C	2.517185000	3.497169211	-0.020625568
C	3.483965000	2.531271921	-0.023713156
C	2.802134000	1.257500600	-0.055570708
C	3.432097000	0.013675609	-0.066962231
C	2.794520000	-1.225054724	-0.096054125
C	3.478243000	-2.497592166	-0.097208571
C	2.514960000	-3.465775412	-0.126159377
C	1.239600000	-2.787429147	-0.140465990
C	0.000000000	-3.424273701	-0.154005643
H	0.000000000	-4.514683033	-0.159545421
H	-2.644016000	-4.545084895	-0.129363464
H	-4.557861000	-2.621594970	-0.073606416
H	-4.522410000	0.008816690	-0.044622878
H	-4.563286000	2.658544225	-0.002260338
H	-2.643832000	4.576592510	0.003250896
H	0.000000000	4.539404996	-0.021947923
H	2.643832000	4.576592510	0.003250896
H	4.563286000	2.658544225	-0.002260338
H	4.522410000	0.008816690	-0.044622878
H	4.557861000	-2.621594970	-0.073606416
H	2.644016000	-4.545084895	-0.129363464

[2]LS

Fe	0.000000000	0.000000000	0.000000000
O	0.000000000	0.000000000	1.812339536
H	0.000000000	0.931998844	2.106784916
N	0.000000000	-0.087339421	-2.094902813
H	0.000000000	-1.053468225	-2.429955289
H	0.829180000	0.386449118	-2.460877799
H	-0.829180000	0.386449118	-2.460877799
N	1.411899000	1.431308385	-0.103134117
N	1.414446000	-1.433199280	-0.020860321
N	-1.411899000	1.431308385	-0.103134117
N	-1.414446000	-1.433199280	-0.020860321
C	-2.777006000	-1.236066600	0.015497275
C	-1.231798000	-2.798087953	0.017536142
C	-2.506708000	-3.474869332	0.067381684
C	-3.465454000	-2.504627320	0.068316058
C	-3.413087000	-0.000377988	-0.002358961
C	-2.777512000	1.234775999	-0.061382627
C	-3.465252000	2.502302023	-0.099643218
C	-2.507489000	3.473054690	-0.163027498
C	-1.232003000	2.799089763	-0.160577026
C	0.000000000	3.442168195	-0.194803707
C	1.232003000	2.799089763	-0.160577026
C	2.507489000	3.473054690	-0.163027498
C	3.465252000	2.502302023	-0.099643218
C	2.777512000	1.234775999	-0.061382627
C	3.413087000	-0.000377988	-0.002358961
C	2.777006000	-1.236066600	0.015497275
C	3.465454000	-2.504627320	0.068316058
C	2.506708000	-3.474869332	0.067381684
C	1.231798000	-2.798087953	0.017536142
C	0.000000000	-3.441404590	0.023959393

H	0.000000000	-4.531064441	0.059825497
H	-2.633702000	-4.553587080	0.104145052
H	-4.545543000	-2.620684626	0.106503482
H	-4.503111000	0.000993774	0.029939890
H	-4.545686000	2.619411126	-0.077252710
H	-2.637431000	4.551308041	-0.203554163
H	0.000000000	4.531642479	-0.235684442
H	2.637431000	4.551308041	-0.203554163
H	4.545686000	2.619411126	-0.077252710
H	4.503111000	0.000993774	0.029939890
H	4.545543000	-2.620684626	0.106503482
H	2.633702000	-4.553587080	0.104145052

Complex **5**, $[Fe^{III}(acac2trien)]^+$

^{6}HS

Fe	0.000000000	0.000000000	0.259485200
O	1.551083100	0.293459600	1.434855000
O	-1.551083100	-0.293459600	1.434855000
N	-0.192299300	2.099661600	0.168038900
N	-1.371314300	0.324711600	-1.488601600
N	0.192299300	-2.099661600	0.168038900
N	1.371314300	-0.324711600	-1.488601600
C	2.125689500	1.371311000	1.847502400
C	1.721037500	2.665919400	1.553316600
C	0.598430200	2.974234600	0.754086900
C	-0.571984200	0.503732000	-2.720084100
C	-2.125689500	-1.371311000	1.847502400
C	-1.721037500	-2.665919400	1.553316600
C	-0.598430200	-2.974234600	0.754086900
C	0.571984200	-0.503732000	-2.720084100
H	-1.184991700	0.404009200	-3.630371500
H	-0.161902400	1.522922600	-2.711745400
H	-1.957251400	-0.510911600	-1.569977400
H	0.372603300	4.043324700	0.619265700
H	2.287034200	3.490715100	1.981976300
H	3.004387400	1.224062100	2.495958300
H	1.184991700	-0.404009200	-3.630371500
H	0.161902400	-1.522922600	-2.711745400
H	1.957251400	0.510911600	-1.569977400
H	-0.372603300	-4.043324700	0.619265700
H	-2.287034200	-3.490715100	1.981976300
H	-3.004387400	-1.224062100	2.495958300
C	-2.205032200	1.481921300	-1.086384500
C	-1.302725400	2.630848200	-0.633735000
C	2.205032200	-1.481921300	-1.086384500
C	1.302725400	-2.630848200	-0.633735000
H	-2.873495300	1.814506600	-1.897322700
H	-2.817176100	1.140288000	-0.242365100
H	-1.903892100	3.350659800	-0.055991100
H	-0.903416000	3.178777400	-1.504671500
H	2.873495300	-1.814506600	-1.897322700
H	2.817176100	-1.140288000	-0.242365100
H	1.903892100	-3.350659800	-0.055991100
H	0.903416000	-3.178777400	-1.504671500

^{2}LS

Fe	0.000000000	0.000000000	0.027637800
O	1.372789700	-0.273382000	1.312129000
O	-1.372789700	0.273382000	1.312129000
N	0.167779300	1.937951800	0.089020700
N	-1.283642700	0.450402500	-1.496620000
N	-0.167779300	-1.937951800	0.089020700
N	1.283642700	-0.450402500	-1.496620000
C	1.931706900	0.648674500	2.027852000
C	1.722263500	2.013445900	1.939784400
C	0.854776300	2.598177100	0.995812200
C	-0.553458500	0.515213100	-2.796781600
C	-1.931706900	-0.648674500	2.027852000
C	-1.722263500	-2.013445900	1.939784400
C	-0.854776300	-2.598177100	0.995812200

C	0.553458500	-0.515213100	-2.796781600
H	-1.237329700	0.373118200	-3.646923000
H	-0.125683800	1.520612700	-2.885362700
H	-2.010506400	-0.271787000	-1.540454500
H	0.747309100	3.690468700	1.016928800
H	2.263310400	2.665951800	2.621537700
H	2.657106800	0.266151200	2.760909500
H	1.237329700	-0.373118200	-3.646923000
H	0.125683800	-1.520612700	-2.885362700
H	2.010506400	0.271787000	-1.540454500
H	-0.747309100	-3.690468700	1.016928800
H	-2.263310400	-2.665951800	2.621537700
H	-2.657106800	-0.266151200	2.760909500
C	-1.900608600	1.762867100	-1.147365900
C	-0.762797300	2.698582100	-0.756243500
C	1.900608600	-1.762867100	-1.147365900
C	0.762797300	-2.698582100	-0.756243500
H	-2.490671800	2.164148600	-1.986289900
H	-2.556577500	1.590327100	-0.287424900
H	-1.163725200	3.571820000	-0.221332700
H	-0.238731600	3.085119900	-1.646978300
H	2.490671800	-2.164148600	-1.986289900
H	2.556577500	-1.590327100	-0.287424900
H	1.163725200	-3.571820000	-0.221332700
H	0.238731600	-3.085119900	-1.646978300

Complex **6**, $[Fe^{IV}(O)(NH_3)_5]^{2+}$

^{5}HS

O	-1.712190000	0.038699000	0.000000000
Fe	-0.110618000	-0.005288000	0.000000000
N	-0.146696000	0.044710000	2.197187000
N	-0.146696000	-2.208428000	0.000000000
N	-0.146696000	0.044710000	-2.197187000
N	2.045180000	-0.104837000	0.000000000
N	-0.007262000	2.206413000	0.000000000
H	0.697227000	0.277109000	2.733040000
H	-0.488151000	-0.841686000	2.587436000
H	0.271577000	-2.674335000	-0.813578000
H	-1.135576000	-2.486922000	0.000000000
H	0.697227000	0.277109000	-2.733040000
H	-0.860710000	0.727139000	-2.479074000
H	0.436402000	2.646123000	0.814869000
H	-0.976816000	2.545894000	0.000000000
H	2.440034000	-0.594678000	-0.811626000
H	2.440034000	-0.594678000	0.811626000
H	-0.860710000	0.727139000	2.479074000
H	0.271577000	-2.674335000	0.813578000
H	2.508409000	0.811621000	0.000000000
H	-0.488151000	-0.841686000	-2.587436000
H	0.436402000	2.646123000	-0.814869000

^{3}LS

O	-1.726284000	0.026304000	0.000000000
Fe	-0.117736000	-0.000785000	0.000000000
N	-0.131418000	0.029962000	2.059929000
N	-0.131418000	-2.060286000	0.000000000
N	-0.131418000	0.029962000	-2.059929000
N	-0.055483000	2.058759000	0.000000000
N	2.063830000	-0.077710000	0.000000000
H	0.665656000	0.455913000	2.547075000
H	-0.254549000	-0.891573000	2.495133000
H	0.293839000	-2.521390000	-0.812524000
H	-1.117084000	-2.351943000	0.000000000
H	0.665656000	0.455913000	-2.547075000
H	-0.963236000	0.556339000	-2.354274000
H	0.383766000	2.502455000	0.814941000
H	-1.029548000	2.386631000	0.000000000
H	2.468359000	-0.564809000	-0.808699000
H	2.468359000	-0.564809000	0.808699000
H	-0.963236000	0.556339000	2.354274000

H	0.293839000	-2.521390000	0.812524000
H	2.531727000	0.836610000	0.000000000
H	-0.254549000	-0.891573000	-2.495133000
H	0.383766000	2.502455000	-0.814941000

Complex **7**, $[Fe^{IV}(O)(NHC)]^{2+}$

^{5}HS

Fe	0.017055000	-0.245714000	0.000000000
O	0.354929000	-1.940004000	0.000000000
C	1.352049000	0.382786000	1.300799000
C	-1.376712000	-0.166322000	-1.449643000
C	-1.376712000	-0.166322000	1.449643000
C	1.352049000	0.382786000	-1.300799000
N	-1.437310000	-0.642802000	2.723612000
N	-2.637774000	0.299817000	1.205783000
N	-2.637774000	0.299817000	-1.205783000
N	-1.437310000	-0.642802000	-2.723612000
N	1.660555000	-0.232816000	-2.466642000
N	2.245133000	1.401645000	-1.188091000
N	2.245133000	1.401645000	1.188091000
N	1.660555000	-0.232816000	2.466642000
C	-2.712187000	-0.477089000	3.254836000
H	-2.966834000	-0.798183000	4.258026000
C	-3.473904000	0.113530000	2.296156000
H	-4.516901000	0.406732000	2.298009000
C	-2.996116000	1.029236000	0.000000000
H	-4.077198000	1.198631000	0.000000000
H	-2.480011000	1.999967000	0.000000000
C	-3.473904000	0.113530000	-2.296156000
H	-4.516901000	0.406732000	-2.298009000
C	-2.712187000	-0.477089000	-3.254836000
H	-2.966834000	-0.798183000	-4.258026000
C	-0.364113000	-1.196321000	-3.576429000
H	-0.245609000	-0.509936000	-4.427626000
H	-0.725891000	-2.157046000	-3.967873000
C	0.975344000	-1.453063000	-2.907931000
H	0.863752000	-2.097186000	-2.022299000
H	1.622042000	-1.957134000	-3.635702000
C	2.726769000	0.400137000	-3.091067000
H	3.134630000	0.064260000	-4.037208000
C	3.105242000	1.431662000	-2.279715000
H	3.897790000	2.162797000	-2.386739000
C	2.288905000	2.247665000	0.000000000
H	1.425832000	2.928076000	0.000000000
H	3.214459000	2.831209000	0.000000000
C	3.105242000	1.431662000	2.279715000
H	3.897790000	2.162797000	2.386739000
C	2.726769000	0.400137000	3.091067000
H	3.134630000	0.064260000	4.037208000
C	0.975344000	-1.453063000	2.907931000
H	1.622042000	-1.957134000	3.635702000
H	0.863752000	-2.097186000	2.022299000
C	-0.364113000	-1.196321000	3.576429000
H	-0.725891000	-2.157046000	3.967873000
H	-0.245609000	-0.509936000	4.427626000

^{3}LS

Fe	0.010878000	-0.165700000	0.000000000
O	0.351006000	-1.765834000	0.000000000
C	1.346836000	0.387887000	1.307511000
C	-1.400635000	-0.181825000	-1.421589000
C	-1.400635000	-0.181825000	1.421589000
C	1.346836000	0.387887000	-1.307511000
N	-1.434424000	-0.623600000	2.714111000
N	-2.675926000	0.265692000	1.203487000
N	-2.675926000	0.265692000	-1.203487000
N	-1.434424000	-0.623600000	-2.714111000
N	1.663206000	-0.228737000	-2.471249000
N	2.260066000	1.392409000	-1.188221000
N	2.260066000	1.392409000	1.188221000

N	1.663206000	-0.228737000	2.471249000
C	-2.696474000	-0.454478000	3.270728000
H	-2.928170000	-0.761502000	4.283841000
C	-3.481802000	0.115159000	2.320075000
H	-4.521652000	0.418383000	2.344911000
C	-3.099082000	0.961072000	0.000000000
H	-4.192280000	1.018285000	0.000000000
H	-2.696840000	1.984280000	0.000000000
C	-3.481802000	0.115159000	-2.320075000
H	-4.521652000	0.418383000	-2.344911000
C	-2.696474000	-0.454478000	-3.270728000
H	-2.928170000	-0.761502000	-4.283841000
C	-0.364669000	-1.179766000	-3.569861000
H	-0.248239000	-0.490460000	-4.418879000
H	-0.733231000	-2.136831000	-3.964537000
C	0.979667000	-1.444267000	-2.920107000
H	0.882710000	-2.109395000	-2.052662000
H	1.617832000	-1.931798000	-3.666653000
C	2.754015000	0.379322000	-3.080258000
H	3.173746000	0.028105000	-4.015458000
C	3.131945000	1.410446000	-2.272072000
H	3.925021000	2.140912000	-2.378776000
C	2.332957000	2.236028000	0.000000000
H	1.509539000	2.963620000	0.000000000
H	3.284175000	2.776939000	0.000000000
C	3.131945000	1.410446000	2.272072000
H	3.925021000	2.140912000	2.378776000
C	2.754015000	0.379322000	3.080258000
H	3.173746000	0.028105000	4.015458000
C	0.979667000	-1.444267000	2.920107000
H	1.617832000	-1.931798000	3.666653000
H	0.882710000	-2.109395000	2.052662000
C	-0.364669000	-1.179766000	3.569861000
H	-0.733231000	-2.136831000	3.964537000
H	-0.248239000	-0.490460000	4.418879000

Complex **8**, $[Fe^{IV}(O)(NHC)(MeCN)]^{2+}$, ^{5}HS

Fe	-0.057235000	0.961656000	0.000000000
O	-0.126200000	2.691132000	0.000000000
C	1.377591000	0.635695000	-1.299454000
C	-1.376001000	0.529259000	1.449525000
C	-1.376001000	0.529259000	-1.449525000
C	1.377591000	0.635695000	1.299454000
N	0.084626000	-3.096906000	0.000000000
N	-1.551251000	0.956667000	-2.730132000
N	-2.459943000	-0.263678000	-1.206215000
N	-2.459943000	-0.263678000	1.206215000
N	-1.551251000	0.956667000	2.730132000
N	1.533710000	1.285522000	2.477072000
N	2.461608000	-0.173815000	1.188460000
N	2.461608000	-0.173815000	-1.188460000
N	1.533710000	1.285522000	-2.477072000
C	-2.725798000	0.439719000	-3.267018000
H	-3.050129000	0.668349000	-4.275184000
C	-3.303533000	-0.327492000	-2.304124000
H	-4.225522000	-0.896309000	-2.308009000
C	-2.593451000	-1.067523000	0.000000000
H	-3.581242000	-1.538182000	0.000000000
H	-1.805885000	-1.837762000	0.000000000
C	-3.303533000	-0.327492000	2.304124000
H	-4.225522000	-0.896309000	2.308009000
C	-2.725798000	0.439719000	3.267018000
H	-3.050129000	0.668349000	4.275184000
C	-0.660729000	1.777931000	3.576411000
H	-0.399506000	1.164209000	4.450964000
H	-1.245572000	2.638109000	3.931346000
C	0.594463000	2.323554000	2.914822000
H	0.346451000	2.924176000	2.026508000
H	1.108367000	2.960551000	3.644545000

C	2.699111000	0.877384000	3.111855000
H	3.017408000	1.278299000	4.066771000
C	3.293152000	-0.041993000	2.294173000
H	4.219362000	-0.592881000	2.404445000
C	2.675630000	-0.996113000	0.000000000
H	1.951760000	-1.825137000	0.000000000
H	3.701145000	-1.376731000	0.000000000
C	3.293152000	-0.041993000	-2.294173000
H	4.219362000	-0.592881000	-2.404445000
C	2.699111000	0.877384000	-3.111855000
H	3.017408000	1.278299000	-4.066771000
C	0.594463000	2.323554000	-2.914822000
H	1.108367000	2.960551000	-3.644545000
H	0.346451000	2.924176000	-2.026508000
C	-0.660729000	1.777931000	-3.576411000
H	-1.245572000	2.638109000	-3.931346000
H	-0.399506000	1.164209000	-4.450964000
C	0.086543000	-4.260521000	0.000000000
C	0.089759000	-5.711648000	0.000000000
H	0.604542000	-6.088610000	-0.894169000
H	-0.941013000	-6.091560000	0.000000000
H	0.604542000	-6.088610000	0.894169000

^{3}LS

Fe	-0.017436000	0.296258000	0.000000000
O	-0.132769000	1.956165000	0.000000000
C	1.460839000	0.302805000	-1.305480000
C	-1.450172000	0.155801000	1.435096000
C	-1.450172000	0.155801000	-1.435096000
C	1.460839000	0.302805000	1.305480000
N	0.026479000	-1.762231000	0.000000000
N	-1.576606000	0.656646000	-2.698995000
N	-2.669561000	-0.417735000	-1.202103000
N	-2.669561000	-0.417735000	1.202103000
N	-1.576606000	0.656646000	2.698995000
N	1.597984000	1.079071000	2.406218000
N	2.666647000	-0.327019000	1.185701000
N	2.666647000	-0.327019000	-1.185701000
N	1.597984000	1.079071000	-2.406218000
C	-2.838816000	0.395041000	-3.226547000
H	-3.129875000	0.722535000	-4.217180000
C	-3.532721000	-0.283918000	-2.281400000
H	-4.545888000	-0.666941000	-2.278332000
C	-3.015418000	-1.149400000	0.000000000
H	-4.097088000	-1.316873000	0.000000000
H	-2.509607000	-2.122651000	0.000000000
C	-3.532721000	-0.283918000	2.281400000
H	-4.545888000	-0.666941000	2.278332000
C	-2.838816000	0.395041000	3.226547000
H	-3.129875000	0.722535000	4.217180000
C	-0.586185000	1.323765000	3.564264000
H	-0.210032000	0.582459000	4.285545000
H	-1.139243000	2.085218000	4.129462000
C	0.570160000	2.011797000	2.866770000
H	0.228061000	2.593223000	2.000226000
H	1.044795000	2.691414000	3.584412000
C	2.859051000	0.935187000	2.977273000
H	3.160259000	1.473770000	3.867588000
C	3.541226000	0.050389000	2.202411000
H	4.548498000	-0.339100000	2.285946000
C	3.028235000	-1.091333000	0.000000000
H	2.515021000	-2.058005000	0.000000000
H	4.109562000	-1.257866000	0.000000000
C	3.541226000	0.050389000	-2.202411000
H	4.548498000	-0.339100000	-2.285946000
C	2.859051000	0.935187000	-2.977273000
H	3.160259000	1.473770000	-3.867588000
C	0.570160000	2.011797000	-2.866770000
H	1.044795000	2.691414000	-3.584412000
H	0.228061000	2.593223000	-2.000226000

C	-0.586185000	1.323765000	-3.564264000
H	-1.139243000	2.085218000	-4.129462000
H	-0.210032000	0.582459000	-4.285545000
C	0.020575000	-2.922617000	0.000000000
C	0.020564000	-4.370873000	0.000000000
H	0.532918000	-4.749085000	-0.895810000
H	-1.010799000	-4.751222000	0.000000000
H	0.532918000	-4.749085000	0.895810000

Complex **9**, $[Fe^{IV}(O)(TMC)(MeCN)]^{2+}$, ^{5}HS

Fe	-0.101122000	-0.278426000	0.000000000
N	-1.497736000	-0.313896000	-1.682244000
N	1.449021000	-0.384783000	-1.620089000
N	1.449021000	-0.384783000	1.620089000
N	-1.497736000	-0.313896000	1.682244000
C	-0.728534000	-1.128012000	-2.670439000
C	0.667649000	-0.570432000	-2.888720000
C	2.257716000	-1.614207000	-1.317436000
C	3.035758000	-1.586344000	0.000000000
C	2.257716000	-1.614207000	1.317436000
C	0.667649000	-0.570432000	2.888720000
C	-0.728534000	-1.128012000	2.670439000
C	-2.738333000	-1.055713000	1.286743000
C	-3.402572000	-0.569225000	0.000000000
C	-2.738333000	-1.055713000	-1.286743000
C	-1.867055000	1.001103000	-2.256460000
C	2.350461000	0.775461000	-1.822146000
C	2.350461000	0.775461000	1.822146000
C	-1.867055000	1.001103000	2.256460000
O	-0.215649000	-1.911678000	0.000000000
H	-2.493996000	0.859104000	-3.150718000
H	-2.430474000	1.584494000	-1.521287000
H	-0.972722000	1.564663000	-2.538313000
H	3.028870000	0.586326000	-2.669292000
H	1.759041000	1.670280000	-2.039251000
H	2.957075000	0.960243000	-0.933663000
H	-2.493996000	0.859104000	3.150718000
H	-0.972722000	1.564663000	2.538313000
H	-2.430474000	1.584494000	1.521287000
H	3.028870000	0.586326000	2.669292000
H	2.957075000	0.960243000	0.933663000
H	1.759041000	1.670280000	2.039251000
H	-2.460488000	-2.110703000	1.173290000
H	-3.449186000	-0.985165000	2.127096000
H	-4.423862000	-0.977326000	0.000000000
H	-3.538818000	0.522722000	0.000000000
H	-3.449186000	-0.985165000	-2.127096000
H	-2.460488000	-2.110703000	-1.173290000
H	-1.262497000	-1.150109000	-3.634653000
H	-0.683978000	-2.150888000	-2.281191000
H	1.221795000	-1.235531000	-3.569614000
H	0.610095000	0.406029000	-3.384549000
H	1.567677000	-2.466135000	-1.326955000
H	2.977208000	-1.747697000	-2.143458000
H	3.646538000	-2.502119000	0.000000000
H	3.773565000	-0.771314000	0.000000000
H	2.977208000	-1.747697000	2.143458000
H	1.567677000	-2.466135000	1.326955000
H	1.221795000	-1.235531000	3.569614000
H	0.610095000	0.406029000	3.384549000
H	-1.262497000	-1.150109000	3.634653000
H	-0.683978000	-2.150888000	2.281191000
N	-0.000487000	1.734562000	0.000000000
C	0.063940000	2.892498000	0.000000000
C	0.144331000	4.333214000	0.000000000
H	-0.351089000	4.737170000	0.894934000
H	-0.351089000	4.737170000	-0.894934000
H	1.197343000	4.650233000	0.000000000

^{3}LS

Fe	-0.065670000	-0.293302000	0.000000000
N	-1.455982000	-0.324948000	-1.575622000
N	1.401864000	-0.378285000	-1.550228000
N	1.401864000	-0.378285000	1.550228000
N	-1.455982000	-0.324948000	1.575622000
C	-0.726046000	-1.102871000	-2.628394000
C	0.646125000	-0.524795000	-2.845974000
C	2.220282000	-1.617910000	-1.305535000
C	3.009792000	-1.625381000	0.000000000
C	2.220282000	-1.617910000	1.305535000
C	0.646125000	-0.524795000	2.845974000
C	-0.726046000	-1.102871000	2.628394000
C	-2.705806000	-1.088972000	1.260252000
C	-3.426689000	-0.646048000	0.000000000
C	-2.705806000	-1.088972000	-1.260252000
C	-1.840852000	1.011576000	-2.101614000
C	2.321479000	0.775136000	-1.750070000
C	2.321479000	0.775136000	1.750070000
C	-1.840852000	1.011576000	2.101614000
O	-0.183587000	-1.929501000	0.000000000
H	-2.495533000	0.888619000	-2.977251000
H	-2.380035000	1.573108000	-1.333121000
H	-0.957037000	1.581634000	-2.397315000
H	2.961861000	0.582573000	-2.624257000
H	1.743268000	1.684727000	-1.931506000
H	2.966288000	0.927474000	-0.885015000
H	-2.495533000	0.888619000	2.977251000
H	-0.957037000	1.581634000	2.397315000
H	-2.380035000	1.573108000	1.333121000
H	2.961861000	0.582573000	2.624257000
H	2.966288000	0.927474000	0.885015000
H	1.743268000	1.684727000	1.931506000
H	-2.418070000	-2.142222000	1.157228000
H	-3.369517000	-1.003225000	2.135725000
H	-4.410476000	-1.137308000	0.000000000
H	-3.646532000	0.431467000	0.000000000
H	-3.369517000	-1.003225000	-2.135725000
H	-2.418070000	-2.142222000	-1.157228000
H	-1.300186000	-1.087770000	-3.568517000
H	-0.666462000	-2.138039000	-2.276198000
H	1.223229000	-1.154183000	-3.539837000
H	0.578180000	0.468977000	-3.304572000
H	1.531939000	-2.469541000	-1.336445000
H	2.927861000	-1.711741000	-2.145990000
H	3.586771000	-2.562528000	0.000000000
H	3.774767000	-0.835505000	0.000000000
H	2.927861000	-1.711741000	2.145990000
H	1.531939000	-2.469541000	1.336445000
H	1.223229000	-1.154183000	3.539837000
H	0.578180000	0.468977000	3.304572000
H	-1.300186000	-1.087770000	3.568517000
H	-0.666462000	-2.138039000	2.276198000
N	0.040934000	1.734807000	0.000000000
C	0.106436000	2.893488000	0.000000000
C	0.185180000	4.335456000	0.000000000
H	-0.310840000	4.739875000	0.894392000
H	-0.310840000	4.739875000	-0.894392000
H	1.237057000	4.655963000	0.000000000

Complex **10**, $[Fe^{IV}(O)^{ax}(PyTACN)(MeCN)]^{2+}$,

^{5}HS

C	-4.004053000	1.463990000	0.094412000
C	-4.682683000	0.303090000	-0.280944000
C	-3.949733000	-0.811551000	-0.690069000
C	-2.559270000	-0.739703000	-0.717087000
N	-1.913417000	0.384112000	-0.332717000
C	-2.614477000	1.466827000	0.055172000
C	-1.688135000	-1.856478000	-1.234025000
N	-0.372496000	-1.846587000	-0.560306000

C	-0.430396000	-2.382866000	0.830796000
C	-0.545990000	-1.274354000	1.872945000
N	0.409264000	-0.157896000	1.598558000
C	0.088269000	0.986990000	2.490529000
Fe	0.169407000	0.324418000	-0.516613000
C	0.714990000	-2.432284000	-1.382273000
C	2.075896000	-2.004925000	-0.841692000
N	2.159545000	-0.520897000	-0.630872000
C	2.925444000	0.118992000	-1.731339000
C	2.741425000	-0.158742000	0.693761000
C	1.832074000	-0.601287000	1.825752000
O	0.072948000	0.457559000	-2.150441000
H	-2.036371000	2.348376000	0.331862000
H	-4.538992000	2.356787000	0.414369000
H	-5.771693000	0.266341000	-0.256936000
H	-4.449948000	-1.730718000	-0.995034000
H	-2.189057000	-2.830996000	-1.122873000
H	-1.516608000	-1.685044000	-2.307626000
H	0.655384000	-3.532660000	-1.407967000
H	0.580225000	-2.056434000	-2.404031000
H	-1.283438000	-3.069229000	0.937384000
H	0.468720000	-2.984714000	1.003969000
H	2.295451000	-2.507651000	0.107676000
H	2.859360000	-2.317226000	-1.545412000
H	-1.553760000	-0.844870000	1.875001000
H	-0.365026000	-1.688012000	2.879103000
H	1.845368000	-1.692146000	1.932027000
H	2.199718000	-0.195037000	2.777802000
H	2.864959000	0.932798000	0.700678000
H	3.744230000	-0.603069000	0.811543000
H	3.964868000	-0.242730000	-1.725812000
H	2.449714000	-0.118566000	-2.688025000
H	2.918957000	1.205898000	-1.598649000
H	0.129995000	0.674494000	3.545095000
H	0.807567000	1.794973000	2.325232000
H	-0.920652000	1.353391000	2.270782000
H	0.640806000	5.140372000	-1.751609000
H	0.795783000	5.526399000	-0.005924000
H	2.236116000	5.031806000	-0.946489000
N	0.688268000	2.379970000	-0.238186000
C	0.896226000	3.493244000	-0.495416000
C	1.155217000	4.875843000	-0.816079000

^{3}LS

C	3.892755000	-0.661178000	-1.360054000
C	4.434741000	-0.720455000	-0.075330000
C	3.606903000	-0.481498000	1.024113000
C	2.261572000	-0.199404000	0.812337000
N	1.739682000	-0.183119000	-0.442216000
C	2.537029000	-0.389490000	-1.510163000
C	1.298318000	0.178901000	1.908440000
N	-0.082744000	-0.203785000	1.507990000
C	-0.329306000	-1.665910000	1.745527000
C	-0.084604000	-2.463626000	0.475587000
N	-0.713200000	-1.768147000	-0.688302000
C	-0.240821000	-2.395260000	-1.954532000
Fe	-0.189782000	0.209680000	-0.541194000
C	-1.152001000	0.653491000	2.096249000
C	-2.433919000	0.473810000	1.290717000
N	-2.171565000	0.565563000	-0.196757000
C	-2.642404000	1.876526000	-0.724504000
C	-2.854478000	-0.529498000	-0.954986000
C	-2.221587000	-1.860496000	-0.616334000
O	-0.298114000	0.404962000	-2.175182000
H	2.052574000	-0.317908000	-2.483447000
H	4.507621000	-0.825102000	-2.243778000
H	5.491295000	-0.944340000	0.071020000
H	4.001849000	-0.507251000	2.039738000
H	1.580778000	-0.272551000	2.871162000
H	1.321185000	1.270708000	2.039388000

H	-1.329625000	0.414039000	3.157093000
H	-0.804194000	1.691784000	2.039963000
H	0.315697000	-2.032768000	2.556244000
H	-1.361406000	-1.787849000	2.092079000
H	-2.894408000	-0.497677000	1.503008000
H	-3.169805000	1.235399000	1.580761000
H	0.986491000	-2.555106000	0.263374000
H	-0.489944000	-3.483277000	0.571823000
H	-2.501880000	-2.188126000	0.390902000
H	-2.572869000	-2.636410000	-1.309157000
H	-2.727341000	-0.295475000	-2.018586000
H	-3.932470000	-0.540351000	-0.727286000
H	-3.740340000	1.921469000	-0.673174000
H	-2.223984000	2.687355000	-0.121444000
H	-2.312177000	1.981060000	-1.762836000
H	-0.536500000	-3.454298000	-1.969917000
H	-0.679479000	-1.865945000	-2.805678000
H	0.849329000	-2.326975000	-2.013831000
H	0.917520000	4.911538000	-1.493420000
H	0.590546000	5.177496000	0.245934000
H	2.202561000	4.605978000	-0.286315000
N	0.305509000	2.077238000	-0.290485000
C	0.675104000	3.173524000	-0.367180000
C	1.121890000	4.542754000	-0.477360000

Complex **11**, $[Fe^{IV}(O)^{eq}(PyTACN)(MeCN)]^{2+}$,

^{5}HS

C	4.003829000	-0.637247000	-1.270910000
C	4.491774000	-0.759062000	0.032315000
C	3.630790000	-0.547255000	1.113587000
C	2.302480000	-0.222326000	0.864632000
N	1.836640000	-0.125489000	-0.409368000
C	2.663944000	-0.318959000	-1.457879000
C	1.300052000	0.118614000	1.936749000
N	-0.071657000	-0.261616000	1.490413000
C	-0.325419000	-1.734037000	1.690433000
C	-0.118255000	-2.542681000	0.416098000
N	-0.795367000	-1.898703000	-0.736372000
C	-0.350729000	-2.487487000	-2.020725000
Fe	-0.152085000	0.247324000	-0.558380000
C	-1.143618000	0.578010000	2.112798000
C	-2.444208000	0.412245000	1.334778000
N	-2.218952000	0.541356000	-0.151807000
C	-2.694079000	1.862330000	-0.647484000
C	-2.887795000	-0.549278000	-0.927328000
C	-2.284298000	-1.913661000	-0.619764000
O	-0.233554000	0.517906000	-2.173895000
H	2.214093000	-0.203533000	-2.444137000
H	4.649542000	-0.788460000	-2.134651000
H	5.536260000	-1.016954000	0.208287000
H	3.989491000	-0.631055000	2.139284000
H	1.554004000	-0.355074000	2.896373000
H	1.311407000	1.208052000	2.089502000
H	-1.291937000	0.314282000	3.172286000
H	-0.802015000	1.618835000	2.065367000
H	0.329735000	-2.117694000	2.484865000
H	-1.351701000	-1.849499000	2.055970000
H	-2.900908000	-0.563587000	1.534976000
H	-3.170846000	1.167174000	1.664448000
H	0.948058000	-2.614977000	0.167971000
H	-0.485543000	-3.572175000	0.568628000
H	-2.559248000	-2.246580000	0.388418000
H	-2.704927000	-2.655797000	-1.313161000
H	-2.755341000	-0.300299000	-1.987824000
H	-3.968108000	-0.557727000	-0.709659000
H	-3.788399000	1.925526000	-0.550535000
H	-2.232098000	2.659159000	-0.057126000
H	-2.407017000	1.975477000	-1.698270000
H	-0.635456000	-3.549958000	-2.076181000
H	-0.806438000	-1.936465000	-2.849747000

H	0.738631000	-2.405277000	-2.103843000
H	0.916755000	5.015486000	-1.762171000
H	0.673185000	5.567494000	-0.075768000
H	2.254194000	4.896164000	-0.577939000
N	0.350419000	2.429054000	-0.056081000
C	0.718763000	3.491655000	-0.347350000
C	1.165950000	4.816771000	-0.709824000

^{3}LS

C	-3.879369000	1.538382000	0.096381000
C	-4.600537000	0.397335000	-0.257037000
C	-3.907928000	-0.740947000	-0.673350000
C	-2.518514000	-0.708022000	-0.724428000
N	-1.823545000	0.401780000	-0.358954000
C	-2.490710000	1.504238000	0.032820000
C	-1.668527000	-1.829705000	-1.242085000
N	-0.336526000	-1.773354000	-0.565126000
C	-0.406867000	-2.346769000	0.830856000
C	-0.551944000	-1.256084000	1.879215000
N	0.384198000	-0.136296000	1.579394000
C	0.046874000	1.031611000	2.431663000
Fe	0.140521000	0.163126000	-0.545699000
C	0.746574000	-2.417577000	-1.375442000
C	2.090647000	-1.952383000	-0.841845000
N	2.096006000	-0.451510000	-0.631857000
C	2.852952000	0.199859000	-1.736972000
C	2.702150000	-0.084465000	0.682494000
C	1.812123000	-0.543262000	1.822061000
O	0.041176000	0.233903000	-2.194448000
H	-1.886694000	2.371993000	0.287634000
H	-4.381525000	2.450253000	0.415704000
H	-5.689425000	0.393584000	-0.213195000
H	-4.439874000	-1.646200000	-0.965116000
H	-2.139460000	-2.813726000	-1.104483000
H	-1.496487000	-1.670263000	-2.316610000
H	0.663439000	-3.514699000	-1.338660000
H	0.605759000	-2.085529000	-2.410196000
H	-1.245544000	-3.052752000	0.895457000
H	0.505626000	-2.928456000	0.998115000
H	2.330362000	-2.438636000	0.111193000
H	2.889539000	-2.228407000	-1.542419000
H	-1.567719000	-0.845089000	1.883126000
H	-0.361758000	-1.671123000	2.883121000
H	1.855533000	-1.632712000	1.938082000
H	2.167841000	-0.120349000	2.771426000
H	2.811978000	1.007077000	0.688333000
H	3.709978000	-0.520618000	0.776961000
H	3.895775000	-0.149439000	-1.726722000
H	2.378331000	-0.047362000	-2.691144000
H	2.830863000	1.285097000	-1.604174000
H	0.102053000	0.757990000	3.496353000
H	0.751903000	1.845344000	2.236068000
H	-0.969290000	1.372366000	2.207950000
H	0.604025000	4.952588000	-1.613466000
H	0.704819000	5.149738000	0.163607000
H	2.172419000	4.745990000	-0.777431000
N	0.576103000	2.044131000	-0.422106000
C	0.804651000	3.175775000	-0.537785000
C	1.088026000	4.583576000	-0.697262000

Complex **12**, $[Fe^{IV}(O)(N4Py)]^{2+}$,

^{5}HS

Fe	-0.234423000	-0.969774000	0.000000000
N	1.368908000	-0.971129000	-1.405082000
N	-1.564569000	-0.298800000	-1.452059000
N	1.368908000	-0.971129000	1.405082000
N	-1.564569000	-0.298800000	1.452059000
N	0.307998000	1.062800000	0.000000000
O	-0.659173000	-2.546737000	0.000000000
C	-3.563293000	-0.456282000	-2.750696000

C	-2.590785000	-1.025101000	-1.936005000
C	-1.460658000	1.017174000	-1.749426000
C	-2.410567000	1.652527000	-2.543686000
C	-3.474685000	0.904144000	-3.052996000
C	-2.590785000	-1.025101000	1.936005000
C	-1.460658000	1.017174000	1.749426000
C	-2.410567000	1.652527000	2.543686000
C	-3.474685000	0.904144000	3.052996000
C	-3.563293000	-0.456282000	2.750696000
C	1.617718000	-1.848702000	-2.389693000
C	2.711113000	-1.688915000	-3.241084000
C	3.552847000	-0.590738000	-3.060972000
C	3.288897000	0.319603000	-2.029911000
C	2.184756000	0.091947000	-1.218697000
C	3.288897000	0.319603000	2.029911000
C	3.552847000	-0.590738000	3.060972000
C	2.711113000	-1.688915000	3.241084000
C	1.617718000	-1.848702000	2.389693000
C	2.184756000	0.091947000	1.218697000
C	-0.220285000	1.708870000	1.242636000
C	-0.220285000	1.708870000	-1.242636000
C	1.810914000	0.919748000	0.000000000
H	-4.377560000	-1.070028000	-3.132958000
H	-2.606762000	-2.075635000	-1.645034000
H	-2.317191000	2.715156000	-2.767814000
H	-4.228437000	1.381207000	-3.679371000
H	-2.606762000	-2.075635000	1.645034000
H	-2.317191000	2.715156000	2.767814000
H	-4.228437000	1.381207000	3.679371000
H	-4.377560000	-1.070028000	3.132958000
H	0.926110000	-2.686670000	-2.475959000
H	2.894991000	-2.418129000	-4.028658000
H	4.411605000	-0.440923000	-3.715202000
H	3.931144000	1.184279000	-1.864780000
H	3.931144000	1.184279000	1.864780000
H	4.411605000	-0.440923000	3.715202000
H	2.894991000	-2.418129000	4.028658000
H	0.926110000	-2.686670000	2.475959000
H	0.556134000	1.637202000	2.019124000
H	-0.402587000	2.779107000	1.071843000
H	0.556134000	1.637202000	-2.019124000
H	-0.402587000	2.779107000	-1.071843000
H	2.320995000	1.893635000	0.000000000

^{3}LS

Fe	-0.098510000	-0.816688000	0.000000000
N	1.341529000	-0.759045000	-1.342966000
N	-1.438582000	-0.378827000	-1.367326000
N	1.341529000	-0.759045000	1.342966000
N	-1.438582000	-0.378827000	1.367326000
N	0.219950000	1.236859000	0.000000000
O	-0.346937000	-2.442170000	0.000000000
C	-3.315330000	-0.882086000	-2.756084000
C	-2.334244000	-1.264437000	-1.848676000
C	-1.480167000	0.916368000	-1.764736000
C	-2.450294000	1.361232000	-2.657793000
C	-3.380193000	0.451375000	-3.162670000
C	-2.334244000	-1.264437000	1.848676000
C	-1.480167000	0.916368000	1.764736000
C	-2.450294000	1.361232000	2.657793000
C	-3.380193000	0.451375000	3.162670000
C	-3.315330000	-0.882086000	2.756084000
C	1.590071000	-1.654116000	-2.313511000
C	2.642832000	-1.464182000	-3.207237000
C	3.439674000	-0.324521000	-3.091852000
C	3.174905000	0.600002000	-2.074459000
C	2.117346000	0.346903000	-1.211964000
C	3.174905000	0.600002000	2.074459000
C	3.439674000	-0.324521000	3.091852000
C	2.642832000	-1.464182000	3.207237000

C	1.590071000	-1.654116000	2.313511000
C	2.117346000	0.346903000	1.211964000
C	-0.376789000	1.803786000	1.243966000
C	-0.376789000	1.803786000	-1.243966000
C	1.728306000	1.173885000	0.000000000
H	-4.019539000	-1.624282000	-3.128731000
H	-2.232170000	-2.284630000	-1.480646000
H	-2.471322000	2.408733000	-2.958271000
H	-4.146175000	0.781053000	-3.864197000
H	-2.232170000	-2.284630000	1.480646000
H	-2.471322000	2.408733000	2.958271000
H	-4.146175000	0.781053000	3.864197000
H	-4.019539000	-1.624282000	3.128731000
H	0.937207000	-2.525395000	-2.341935000
H	2.830651000	-2.208406000	-3.979629000
H	4.263601000	-0.154474000	-3.784460000
H	3.780489000	1.497932000	-1.955723000
H	3.780489000	1.497932000	1.955723000
H	4.263601000	-0.154474000	3.784460000
H	2.830651000	-2.208406000	3.979629000
H	0.937207000	-2.525395000	2.341935000
H	0.410858000	1.872279000	2.009060000
H	-0.738297000	2.826683000	1.070160000
H	0.410858000	1.872279000	-2.009060000
H	-0.738297000	2.826683000	-1.070160000
H	2.205068000	2.164221000	0.000000000

References

1. Phung, Q. M.; Feldt, M.; Harvey, J. N.; Pierloot, K. Toward Highly Accurate Spin State Energetics in First-Row Transition Metal Complexes: A Combined CASPT2/CC Approach. *J. Chem. Theory Comput.* **2018,** *14*, 2446-2455.
2. Radoń, M. Benchmarking quantum chemistry methods for spin-state energetics of iron complexes against quantitative experimental data. *Phys. Chem. Chem. Phys.* **2019,** *21*, 4854-4870.
3. Phung, Q. M.; Martín-Fernández, C.; Harvey, J. N.; Feldt, M. Ab Initio Calculations for Spin-Gaps of Non-Heme Iron Complexes. *J. Chem. Theory Comput.* **2019,** *15*, 4297-4304.
